# Benchmarking Class Imbalance Mitigation Strategies Across Deep CNN Architectures for Skin Cancer Classification

**DOI:** 10.3390/diagnostics16162571

**Published:** 2026-08-14

**Authors:** Irshad Ahmad, Muhammad Khubaib, Saleh M. Altowaijri

**Affiliations:** 1 Computer Science Department, Islamia College Peshawar, Peshawar 25120, Pakistan; cskhubaib@gmail.com; 2 Department of Information Systems, Faculty of Computing and Information Technology, Northern Border University, Rafha 91911, Saudi Arabia

**Keywords:** skin lesion classification, class imbalance, ISIC 2019, deep learning, pretrained CNN models, imbalance mitigation, Balanced MixUp, SMOTE, medical image analysis

## Abstract

**Background/Objectives**: Class imbalance is one of the major challenges in automated skin lesion classification since the number of categories of malignant and clinically significant skin lesions is normally less than the benign ones. However, due to this imbalance, deep convolutional neural networks (CNNs) tend to overlook minority classes and fail to recognize them with an acceptable accuracy, which leads to a decrease in diagnostic reliability. A wide range of imbalance mitigation techniques has been suggested, but their effectiveness is found to differ significantly depending on CNN architecture, and detailed comparative studies of these techniques for a consistent experimental setup are still limited. **Methods**: This study proposes a comprehensive benchmarking framework that tests sixteen class imbalance mitigation methods by applying them to six pretrained CNN architectures—EfficientNet-B0, EfficientNet-B3, ResNet50, DenseNet121, InceptionV3 and MobileNetV2—on the official ISIC 2019 skin lesion dataset. The tested techniques are conventional resampling techniques, synthetic sample generation techniques, algorithm-level learning techniques, data augmentation techniques, and hybrid techniques. The dataset was partition into a separate training set and testing set, and stratified cross-validation was only conducted on the training set to ensure the study was fair and reproducible. Both models have been optimized with the same optimizer, learning rate, batch size, epochs and preprocessing pipeline. The performance of the models was evaluated by computing the accuracy, precision, recall and F1-score. **Results**: The experimental results show that the effect of class imbalance mitigation is very specific to the underlying CNN architecture. The traditional undersampling and oversampling methods yielded only moderate improvements, while feature space and hybrid methods yielded more consistent results. When coupled with EfficientNet-B3, Balanced MixUp improved the overall performance of the model by achieving an accuracy of 92.39%, an increase in precision of 93.3%, a recall of 91.36%, and an F1-score of 92.33%. However, some architectures such as ResNet50 performed better with iterative learning techniques, such as Cumulative Learning and Yielding Multi-Fold Training, which suggests that there is a diversity in how different network architectures react to imbalance mitigation methods. **Conclusions**: This paper highlights the importance of selecting appropriate technique–architecture combinations for addressing long-tailed data distributions in medical imaging. The proposed benchmarking framework provides valuable insights for developing robust and reliable deep learning systems for skin lesion classification and other medical imaging tasks affected by severe class imbalance.

## 1. Introduction

One of the most important issues in supervised learning is class imbalance, which has posed a big problem in medical image classification for developing reliable models using deep learning. In many real-world medical datasets, the incidence of diseases is naturally skewed, with many common diseases having many training samples, while clinically important diseases are only represented by a small number of images. This imbalance results in a very high optimization bias towards majority classes that negatively affects the learning of minority-class characteristics, as well as diagnostic performance in deep learning models. The wrong diagnosis of rare but clinically important diseases may cause a delay in diagnosis, influence the treatment strategy, and have a negative impact on patient outcomes, as is the case in medical image analysis [1,2,3,4].

Automated skin lesion classification is one of the most important medical imaging applications that has gained much attention recently because of the escalating diagnosis rate of skin cancer globally. World cancer statistics show that skin cancer is one of the most commonly diagnosed types of cancer, and early detection significantly increases patient survival and the effectiveness of treatment [5]. Dermoscopic imaging has emerged as an essential noninvasive skin diagnosis tool that allows the dermatologist to see the structures of the lesions in a clearer manner than a regular visual examination. In recent years, great efforts have been made in the field of deep learning to utilize CNN to learn highly discriminative image features directly from dermoscopic images, without using handcrafted feature [6,7,8,9]. Even though some pretrained models like ResNet50, DenseNet121, EfficientNet, InceptionV3, MobileNetV2 and VGG have shown remarkable performance in the classification task for benchmark datasets like ISIC, their performance drops significantly on highly imbalanced datasets where the optimization process is dominated by the majority-class sample [9,10,11,12].

However, in recent years, many class imbalance mitigation strategies have been proposed to overcome the above limitation. There are basically three types of approaches: data-level, algorithm-level and hybrid. Data-level methods change the distribution of the classes prior to training the model, either by adding more instances of minority classes or by removing instances of majority classes. The methods that are widely used are Random Oversampling (ROS), Random Undersampling (RUS), the Synthetic Minority Oversampling Technique (SMOTE), Adaptive Synthetic Sampling (ADASYN) and SMOTE-LOF, which enhance class representation by employing different sampling strategies [5,13,14,15,16]. The techniques are easily adopted and have been shown to yield performance gains across a wide range of medical image classification applications, though it is easy to see that oversampling could lead to including redundant information, while undersampling could lead to a loss of training samples.

The aim of algorithm-level approaches is to modify the optimization objective instead of changing the distribution of the original data, thus handling class imbalance. Cost-Sensitive Learning is a learning method that gives more weight to misclassifications of minority classes, thereby forcing the model to learn a well-balanced boundary during the learning process [7]. In the same way, Focal Loss minimizes the impact of well-defined samples belonging to the majority class and emphasizes the learning of more challenging examples or those belonging to the minority class, hence being especially recommendable for classification tasks with significant class imbalance [8,9,10,11,12,13,14,15]. With recent optimization strategies (curriculum learning, Cumulative Learning and class-balanced optimization), the learning stability and generalization improvements under long-tailed data distribution have been enhanced [16,17].

More recently, researchers have suggested more sophisticated techniques to mitigate imbalance in data, feature space learning and ensemble optimization. GraphSMOTE is an extension of the traditional SMOTE which creates synthetic samples in the graph embedding space, maintaining neighborhood relations among minority samples [18]. Balanced MixUp enhances feature interpolation in a class-aware fashion, and has shown promising performance in various computer vision tasks [19]. Minority-class representation has been improved with other techniques, such as class decomposition using CDSMOTE, Pseudo-Labeling, augmentation using GAN and the introduction of ensemble learning and Yielding Multi-Fold Training, which further improved model robustness and generalization [12,13,14,18]. Each of these methods has shown a certain degree of success, but their success is dependent on the characteristics of the dataset, network architecture, and training method.

Although numerous imbalance mitigation approaches have been proposed and studied in the literature, most of the studies test one or two methods with just one CNN architecture, without considering other deep learning models to see if a specific balancing strategy always works well for them. The effectiveness of an imbalance mitigation technique is expected to vary widely depending on the underlying CNN architecture because the architecture varies widely in the number of layers in the network, the features they extract, how efficient they are in terms of their parameters, and how they are optimized. For instance, lightweight networks like MobileNetV2 can be better trained using feature space augmentation than deeper networks like EfficientNet-B3 and DenseNet121, and residual networks like ResNet50 can be better trained using iterative learning methods than synthetic oversampling. In the current literature, however, a systematic benchmarking study that compares several imbalance mitigation techniques between different CNN architectures and in similar experimental settings is missing [15,17,18,20].

To tackle this research gap, this study proposes a comprehensive benchmarking framework to test sixteen of the most common class imbalance mitigation techniques on six pretrained CNN architectures using the official ISIC 2019 skin lesion dataset [19,20,21]. The architectures studied are EfficientNet-B0, EfficientNet-B3, ResNet50, DenseNet121, InceptionV3, and MobileNetV2, while the imbalance mitigation techniques explored are traditional resampling techniques, synthetic sample generation techniques, algorithm-level learning techniques, feature space augmentation techniques, and hybrid techniques. To enable fair, reproducible and unbiased comparisons among all architecture–technique combinations, all experiments were performed with a common experimental protocol and identical preprocessing, optimization and evaluation protocols.

The following are the key findings of this study:

- A detailed benchmarking framework is suggested to perform the comprehensive evaluation of sixteen class imbalance mitigation techniques on six popular pretrained CNN architectures using the official ISIC 2019 skin lesion dataset in a systematic way.
- A unified experimental protocol is set up, where all the models are trained with the same preprocessing techniques, optimization parameters, training setups and evaluation methods for fair and reproducible comparisons between different imbalance mitigation strategies.
- A comparative performance analysis is used to explore the efficiency of all three imbalance mitigation methods at the data level (DLIM), the algorithm level (ALIM), and a hybrid level (HLIM) on different CNN architectures to gain insights into their performance strengths and weaknesses in highly imbalanced medical image classification tasks.
- Through extensive experimental evaluation, it is shown that the effectiveness of class imbalance mitigation is heavily reliant not only on the type of balancing strategy, but also on the underlying CNN architecture. Balanced MixUp with EfficientNet-B3 performed best overall in terms of classification accuracy, while other approaches yielded improved results with different methods like SMOTE, Cumulative Learning and Yielding Multi-Fold Training. The findings offer a practical solution for the selection of imbalance mitigation techniques based on architecture used for automated skin lesion classification.

## 2. Related Work

Class imbalance is one of the most well-studied problems in machine learning and deep learning, as it greatly impacts the performance of classification models, especially in medical image analysis. In skin lesion classification, minority classes may represent clinically relevant diseases with a few training samples in comparison to the other disease classes considered benign. In this way, deep learning models usually learn a decision boundary biased towards the majority classes, leading to poor recognition for rare but clinically important lesion [6,18,22]. Because of this, many imbalance mitigation strategies have been proposed, each with different underlying principles and implementation complexities and varying effectiveness.

Data-level methods, algorithm-level methods and hybrid methods can be broadly classified as existing class imbalance mitigation methods. Data-level methods try to rebalance the dataset prior to model training through resampling, synthetic sample generation, or data augmentation. Algorithm-level techniques alter the learning process by adding class-dependent loss functions or optimization methods that focus on learning minority classes, but not on the distribution of the data itself. Hybrid methods are a blend of several methods, merging the strengths of data-level and algorithm-level methods, resulting in better classification performance when the dataset contains many imbalanced classes [6,18,22,23,24].

In recent years, some advanced deep learning techniques, such as GraphSMOTE, Balanced MixUp, GAN-based augmentation methods, curriculum learning, Pseudo-Labeling and ensemble learning, have also been adopted for solving the problem of imbalance. They have been shown to be successful in several computer vision and medical image classification tasks for increasing the representation of minority classes, adding diversity to features, and decreasing the model’s bias toward majority classes, respectively [6,12,25,26]. Most of the previous studies assess a limited number of balancing techniques with a single deep learning architecture, however, and it is difficult to know whether a specific imbalance mitigation technique is consistently effective for different convolutional neural network (CNN) models.

The following subsections summarize the relevant literature in the fields of data-level, algorithm-level and hybrid approaches for class imbalance mitigation, explaining the pros and cons of each approach and their suitability for medical image classification.

### 2.1. Data-Level Strategies

Data-level methods are among the most popular methods for coping with an imbalanced class distribution; they alter the class distribution prior to training the model, but do not alter the learning algorithm. They aim to increase the representation of minority classes either by increasing the number of minority samples or by decreasing the majority classes. These approaches are independent of the architecture of the classifiers, are widely used in medical image classification problems [18,22], and can be easily added to various machine learning and deep learning architectures.

Random Oversampling (ROS) and Random Undersampling (RUS) are the most widely used data-level techniques. Randomly amplifying minority classes with duplicate copies to make them have a similar number of training examples as the majority classes is called Random Oversampling. While ROS is easy to implement, and it increases the representation of minority classes, it also can suffer from overfitting if the same sample is duplicated many times [18,22,27]. On the other hand, in RUS, the samples of the majority class are removed at random, thereby lowering the computational burden, but at the same time valuable information which can enhance model performance may be lost.

However, these disadvantages have been addressed by several proposed synthetic oversampling methods. The Synthetic Minority Oversampling Technique (SMOTE) is a technique that creates new minority samples by interpolating between adjacent minority samples instead of creating copies of existing minority samples, thereby providing more diversity in the samples and avoiding overfitting [23,28]. Based on SMOTE, ADASYN dynamically creates more synthetic samples for hard minority samples in areas close to decision boundaries, which will enhance the capability of the classifier to learn hard samples [5]. Likewise, SMOTE-LOF uses SMOTE and a Local Outlier Factor (LOF) algorithm to prevent the creation of synthetic samples around noisy or unreliable data instances, which leads to more representative minority samples [12]. More recently, GraphSMOTE has been proposed to enhance this concept by synthesizing graph samples in the graph embedding space with the preservation of neighbor relationships, which has proven to be effective in graph-based classification tasks [25] and has shown promising results.

Another method for addressing class imbalance is image augmentation. Parameters such as rotation, flipping, scaling, cropping and color jittering are widely used augmentation techniques which help model generalization and mitigate overfitting by adding diversity to training data while maintaining class labels [29]. Feature space augmentation has also been explored recently, where synthetic samples are created based on high-level feature representations, resulting in more discriminative features while reducing the amount of unrealistic artifacts [26].

Recent work includes Balanced MixUp, which is particularly effective for handling highly imbalanced data. Balanced MixUp is a class-aware MixUp which tries to sample minority-class samples with a higher probability during image mixing compared to the conventional MixUp method. This approach creates smoother decision boundaries, increases the diversity of features and improves the recognition of minority classes [30]. A better approach is CDSMOTE, which first divides each class into homogeneous sub-clusters and then uses synthetic oversampling, thus creating more representative synthetic samples, which leads to better local decision boundaries [31].

Data-level imbalance mitigation has been strengthened by generative models based on Generative Adversarial Networks (GANs), which have been used to create realistic synthetic medical images of minority classes, such as DCGAN, Conditional GAN (cGAN), BSS-GAN and DB-cGAN [24,32,33]. GAN-generated images have shown promising results when applied to medical image classification tasks, such as skin lesion classification, by enhancing the diversity of features in an image and decreasing the class imbalance. Moreover, hybrid GAN–CNN models have been shown to have better classification capabilities in various highly imbalanced applications such as anomaly detection, fraud detection, or intrusion detection [34,35].

At the data level, approaches are still promising due to their simplicity, efficiency and adaptability to various deep learning architectures. The performance of these approaches, however, is dependent on both the type of data and the CNN architecture. However, for skin lesion classification, it is necessary to have a comprehensive benchmarking study to determine the best data-level imbalance mitigation approach.

### 2.2. Algorithm-Level Strategies

Algorithm-level approaches address class imbalance by modifying the learning process instead of changing the original data distribution. Unlike data-level methods, these techniques improve minority-class learning by adjusting the optimization objective, loss function, or training strategy while preserving the original dataset. Because the training data remain unchanged, algorithm-level methods avoid the risk of generating unrealistic synthetic samples and can be easily integrated into existing deep learning architecture [6,17].

One of the most widely adopted algorithm-level techniques is Cost-Sensitive Learning, which assigns higher penalties to the misclassification of minority-class samples during model training. By increasing the classification cost of rare classes, the optimization process encourages the model to learn more balanced decision boundaries and reduces the bias toward majority classes [6]. Cost-Sensitive Learning has been successfully applied to numerous medical image classification problems where minority-class recognition is clinically important.

Another important category consists of loss function-based methods, including Focal Loss, Class-Balanced Loss, and other dynamically weighted loss functions. These methods improve learning by assigning greater importance to difficult or underrepresented samples during optimization. Among them, focal loss reduces the contribution of easily classified majority-class samples while focusing training on hard examples, making it particularly effective for long-tailed image classification tasks [7,36]. Similarly, Class-Balanced Loss incorporates an effective number of samples for each class, enabling more balanced optimization across different class distributions.

More recently, advanced loss functions such as Influence-Balanced (IB) Loss have been proposed to further improve learning from imbalanced datasets. IB Loss dynamically adjusts sample weights according to their influence during optimization, reducing the dominance of majority-class samples while preserving informative minority examples. Previous studies have reported that IB Loss outperforms conventional loss functions, including Focal Loss and LDAM, on several highly imbalanced benchmark datasets [37].

Algorithm-level methods also include curriculum-based learning strategies, which gradually modify the learning process throughout training. One example is the Cumulative Learning Strategy (CLS)**,** where the model progressively learns from increasingly difficult samples or receives greater exposure to minority-class examples as training proceeds. This gradual learning process improves optimization stability, reduces overfitting, and enhances the model’s ability to generalize to minority classes [31].

In addition to optimization-based techniques, ensemble learning has become an effective algorithm-level solution for handling class imbalance. Methods such as Balanced Bagging, RUSBoost, and Balanced Random Forest construct multiple classifiers using different balanced subsets of the training data and combine their predictions to improve robustness [9]. By integrating the outputs of multiple learners, Ensemble Methods reduce model variance, improve minority-class recognition, and often achieve better generalization than single-model approaches.

Overall, algorithm-level techniques provide an effective alternative to data-level methods because they directly optimize the learning process without modifying the original dataset. However, their effectiveness depends on the choice of loss function, weighting strategy, and network architecture. Consequently, a comprehensive evaluation across multiple pretrained CNN architectures is necessary to determine which algorithm-level strategies provide the most reliable performance for highly imbalanced skin lesion classification.

### 2.3. Hybrid Approaches

Hybrid approaches use a fusion of the advantages of both data-level and algorithm-level imbalance mitigation techniques to boost the classification accuracy on highly imbalanced datasets. These methods do not necessarily use a single approach, but rather combine multiple techniques such as synthetic sample generation, cost-sensitive optimization, advanced loss functions and ensemble learning to improve minority-class representation whilst ensuring overall model generalization. Hybrid methods are one of the most promising research paths in medical image classification due to their ability to simultaneously tackle the two key issues of data imbalance and optimization bias [16,24,28].

A hybrid approach integrating data augmentation with deep CNN classifiers and Cost-Sensitive Learning by using GANs is widely used. These methods involve using Generative Adversarial Networks (GANs) to create synthetic images of the minority classes, while adjusting the loss function used to train the classifier or optimizing it for the minority classes to enhance the system’s ability to recognize them. In the past, the combination of GAN-produced images and CNN-based classifiers has been proven to enhance the classification accuracy over the augmentation and Cost-Sensitive Learning methods alone, especially in the medical image analysis field where labeled data is scarce [11,33].

New developments have also led to the introduction of semi-supervised learning methods, such as Pseudo-Labeling and FixMatch, which are able to leverage significant amounts of unlabeled data to enhance model performance. These techniques can be used to obtain reliable pseudo-labels for unlabeled samples and integrate pseudo-labels into the training process through confidence-based selection or class-aware threshold. Semi-supervised learning boosts minority-class representation by adding informative training samples and decreases the amount of manual annotations required [11,13].

Ensemble-based training approaches, such as stratified cross-validation, ensemble averaging, snapshot ensembling, and temporal ensembling including Yielding Multi-Fold Training (Y-MuFT) [28,36], are often used in hybrid frameworks to boost the robustness of the model. These methods help to decrease the variance of the predictions made by the models by combining different trained models or multiple training stages, which leads to more stable predictions and better results for minority classes. In highly imbalanced medical image classification problems, ensemble techniques have been shown to be reliable and to decrease model variance, leading to improvements in the accuracy of model predictions.

Overall, hybrid approaches offer a complete solution to the problem of class imbalance, combining complementary data-level and algorithm-level approaches in a single learning framework. These strategies normally outperform individual imbalance mitigation strategies, but their effectiveness relies on the combination of augmentation methods, optimization strategies and CNN architectures used. Thus, it is crucial to perform systematic benchmarking to determine the best hybrid approach to automated skin lesion classification for very imbalanced data.

## 3. Proposed Framework

**Figure 1:**
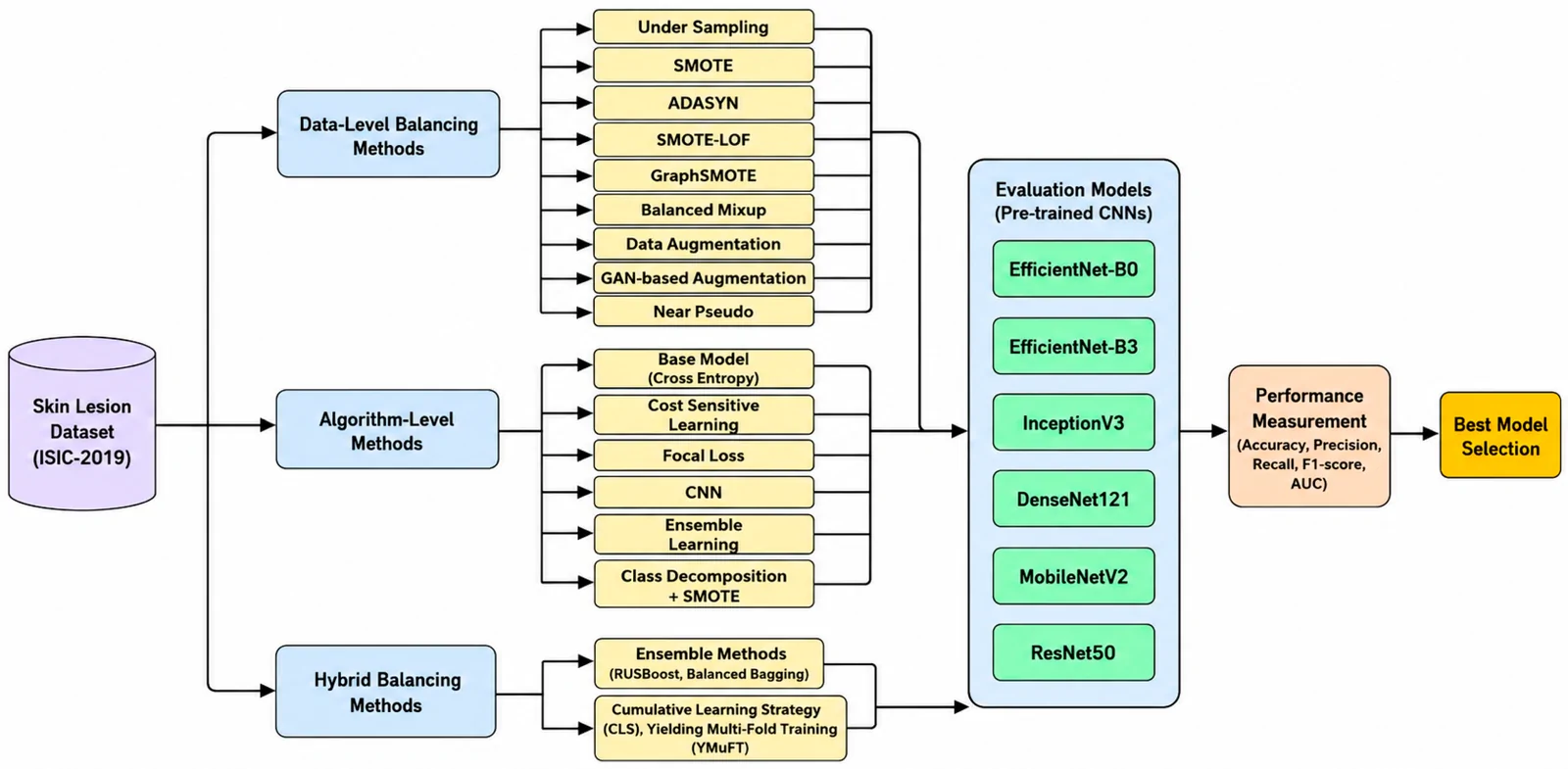
Diagram of the application of the balancing techniques to various pretrained models.

The aim of this study is to present a single framework for benchmarking the performance of various class imbalance mitigation approaches for skin lesion classification in a systematic way. Figure 1 shows the overall workflow of the proposed framework. The proposed framework has four major steps: dataset preparation, imbalance mitigation, CNN model training, and performance evaluation.

The first step was preparing and organizing the dataset of skin lesions from the ISIC 2019 competition for model development. The training data was then preprocessed, various imbalance mitigation techniques were applied, and the original testing data was kept, measuring the performance without any bias. For a thorough comparison, the analyzed techniques were classified into three groups: data-level techniques, algorithm-level techniques, and hybrid techniques.

Next, six pretrained CNN architectures (EfficientNet-B0, EfficientNet-B3, ResNet50, DenseNet121, InceptionV3, and MobileNetV2) were trained using the balanced training datasets. The same preprocessing pipeline, an optimizing process and evaluation protocol was used for each network, which ensures that the performance difference is attributed to the applied imbalance mitigation technique and not to any differences in network configurations.

After the model training, the performance of each architecture was assessed with commonly used classification metrics, such as accuracy, precision, recall, and F1-score. The experimental results for all imbalance mitigation techniques were then compared to find out the working efficiency of various CNN architectures. Comparative evaluation allows us to identify the architecture-specific methods of imbalance mitigation, as opposed to expecting a perfect performance of one balancing method for all deep learning models.

The proposed framework was conceived to enable a fair, repeatable and systematic assessment of methods to mitigate imbalance in a comparable experimental setup. The trained system was evaluated in the same way in all experiments, and the comparison’s objective was to compare different balancing strategies; moreover, practical insights can be gained in selecting appropriate balancing strategies for highly imbalanced skin lesion classification tasks.

To ensure reproducibility, all six CNN architectures within the proposed framework were fine-tuned with a common, fixed training configuration: the Adam optimizer with an initial learning rate of 1 × 10^−4^ and no learning rate scheduler, a batch size of 32, and a fixed budget of 10 training epochs with no early stopping criterion (i.e., all models were trained for the full 10 epochs regardless of validation performance). Input dermoscopic images were resized to 224 × 224 pixels and normalized using ImageNet mean and standard deviation statistics, with random horizontal flipping, random rotation (±10°), and color jitter applied as preprocessing/augmentation. Cross-entropy loss was used for all models, and training was performed under 3-fold stratified cross-validation on the 80% training split, with the independent 20% test split held out until final evaluation. The complete parameter set is summarized in Table 1.

Figure 1 shows that each method of mitigating imbalance has been applied separately to create separate training datasets. The mentioned datasets were then utilized to fine-tune six pretrained CNNs, namely, EfficientNet-B0, EfficientNet-B3, InceptionV3, DenseNet121, ResNet50, and MobileNetV2. To maintain a uniform experimentation environment, all the models were fine-tuned applying the same preprocessing techniques, same optimization parameters, same learning rate schedules, and same batch sizes.

Model performance was analyzed by computing classification metrics such as accuracy, precision, recall, and F1-score. These metrics offer additional information about the classification capabilities of each model, especially in the case of an imbalanced multi-class dataset, where accuracy does not reflect classification results for minority classes. Macro-averaged evaluation metrics were considered to ensure the equal contribution of each lesion category to the evaluation process regardless of its frequency.

This comparison was performed to find out how the imbalance mitigation techniques influence the performance of the CNN architecture. Evaluation of every architecture in the same experimental environment allows us to assess architecture–technique compatibility and to identify the most appropriate combinations offering reliable classification performance for skin lesion recognition.

## 4. Experimental Results

In this section, the experimentally obtained results of the suggested benchmarking methodology for skin lesion classification based on the ISIC 2019 dataset are provided. The aim is to evaluate the efficiency of sixteen class imbalance mitigation approaches when used in conjunction with six pretrained convolutional neural network (CNN) architectures under the same experimental paradigm. All models were trained under the same conditions to ensure a fair comparison of the balancing strategies investigated.

Model performance was measured using performance indicators such as accuracy, precision, recall, and F1-score. These measures allow for an integrated analysis of a classifier’s ability to classify data into multiple classes with an uneven number of samples in those classes. While the accuracy indicator allows us to measure the global classification capabilities of a model, precision, recall, and F1-score help to estimate its recognition abilities regarding minority and majority classes. It ensures a more balanced analysis of the methods investigated rather than the analysis of overall prediction accuracy only.

Below, the comparative analysis of each of the six pretrained CNN architectures is provided. For each model, the impact of various imbalance mitigation methods is analyzed and compared to determine the best balancing approach under the same experimental conditions. The results are provided through quantitative performance measures along with comparative tables.

### 4.1. Dataset Description

The experimental validation was carried out on the ISIC 2019 skin lesion dataset [20,21,37], which consists of 25,331 dermoscopic images in eight categories. The chosen dataset offers an appropriate benchmark for the analysis of the impact of class imbalance on deep learning models, as it is characterized by a high level of class imbalance. Table 1 shows the number of images and the proportion of samples per lesion category in this dataset.

Table 1 shows that the distribution of images across lesions is quite imbalanced. Thus, melanocytic nevus (NV) forms the largest category, accounting for 50.83% of the images, while dermatofibroma (DF) and vascular lesions (VASC) consist of 239 (0.94%) and 253 (1.00%) images, respectively. In turn, actinic keratosis (AK) and squamous cell carcinoma (SCC) have 867 (3.42%) and 628 (2.48%) images, respectively.

**Figure 2:**
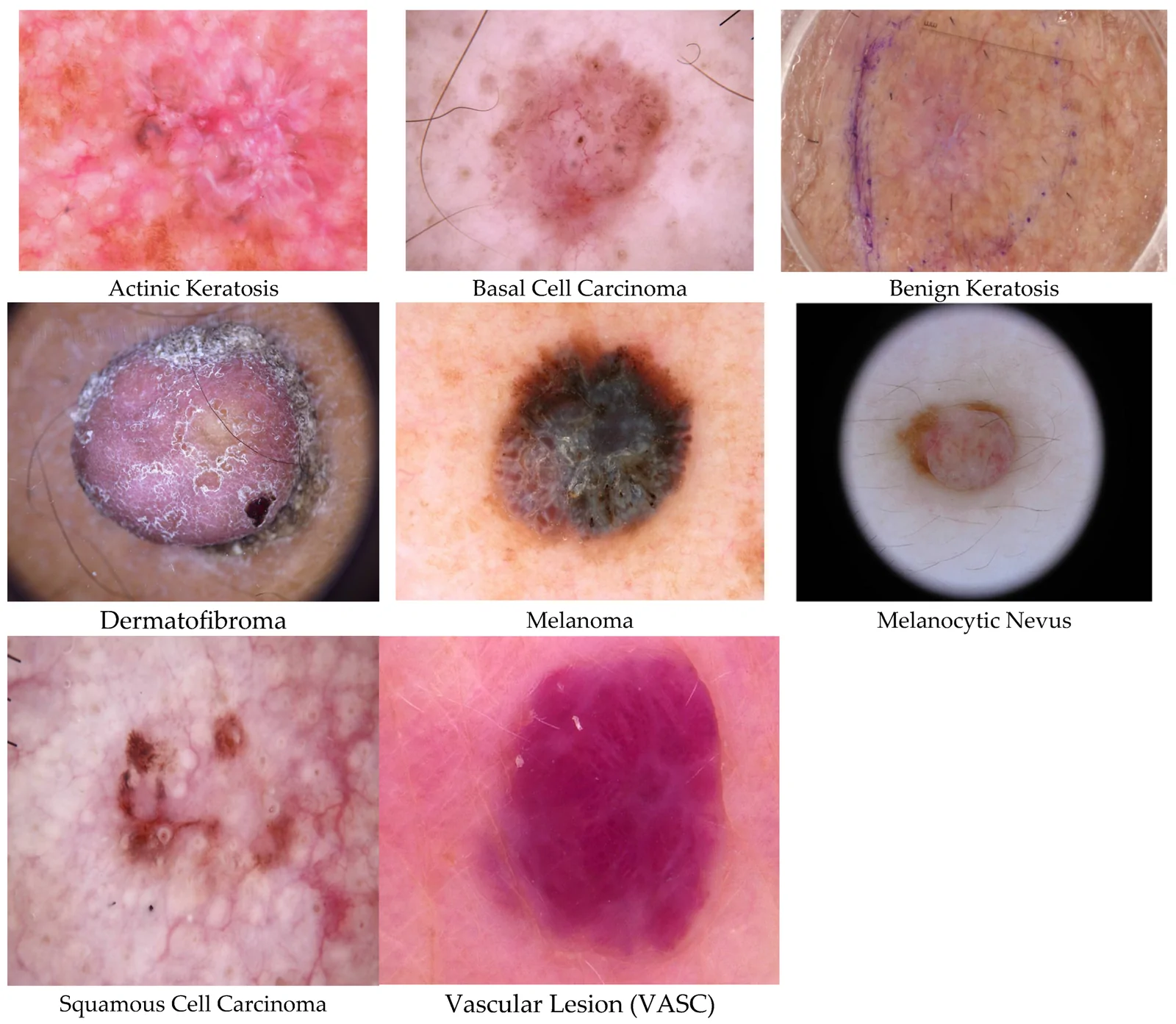
Example images of dermoscopic of lesions in the eight classes of the ISIC 2019 dataset.

Such a class imbalance serves as an adequate benchmark for examining the effectiveness of class imbalance mitigation approaches. Due to the relatively small number of images in minority classes, it becomes harder for deep learning networks to classify the data correctly. Therefore, further experiments (presented in the next sections) analyze the effect of various balancing techniques on pretrained CNNs. Some sample images from the various classes are shown in Figure 2.

To systematically assess the efficacy of the various techniques used to handle class imbalance problems in skin lesion classification, sixteen different balancing techniques were examined in the current research. These techniques can be classified into three categories: data-level, algorithm-level and hybrid methods. Each of these techniques was separately used on the training set before fine-tuning the six CNN architectures introduced in Section 3.

The techniques used in this study are resampling methods, synthetic sample creation methods, loss optimization methods and hybrid learning methods. The use of these methods under similar experimental conditions allowed an unbiased comparison of their efficiency in relation to different CNN architectures.

All the CNN architectures were trained using the same preprocessing pipeline, optimization criteria, learning rate scheduling and data split method. This way, it could be ensured that the difference in performance was due to the balancing methods investigated rather than the particularities of the training process itself. The comparative analysis of each pretrained CNN architecture is given in the next subsections.

### 4.2. Experimental Setup

Experiments were carried out based on the ISIC 2019 skin lesion dataset using the approach discussed in Section 3. At the beginning, the dataset was split into 80% training and 20% independent test subsets. The latter was only used in the final experiment and was excluded from any use during the training of the models and hyperparameter tuning. To increase the reliability of the model’s evaluation and decrease the variability in their performance, 3-fold stratified cross-validation was performed for the training subset. Specifically, for each fold, two subsets were used for the model training, and the rest were used for validation. After the cross-validation procedure was completed, the best-performing model according to the validation results was chosen and was evaluated on the independent test subset to obtain the final classification results.

#### 4.2.1. Dataset Splitting and Cross-Validation Protocol

All class imbalance mitigation techniques were applied only to the training folds, not to the validation fold, and never to the held out 20% test set, to prevent information leakage between synthetic minority samples and evaluation data. Within the 80% training set, each imbalance mitigation technique was reapplied independently within each fold’s training portion during 3-fold stratified cross-validation. The best-performing model checkpoint across folds, selected using the validation F1-score, was evaluated once on the independent test set to produce the reported results. The reported ± values denote the standard deviation across the three cross-validation folds.

To carry out a fair comparison between all investigated methods, an identical training setup was kept for all experiments. Each imbalance mitigation approach was separately applied to the training data, and then the pretrained CNN models were tuned on it. For each architecture and each imbalance mitigation method, the same optimizer, learning rate, batch size, preprocessing pipeline, data augmentation strategy, and loss function were used. Therefore, the differences in the results obtained by the different approaches could only be due to the differences in the imbalance mitigation approaches investigated and not the training configuration.

All experiments used six pretrained convolutional neural networks (CNNs): EfficientNet-B0, EfficientNet-B3, ResNet50, DenseNet121, InceptionV3, and MobileNetV2. All the architectures were trained under the same experimental protocol and evaluated in identical conditions to ensure the objective comparison of the models. The imbalance mitigation approaches under investigation were categorized as data-level, algorithm-level, and hybrid approaches. Each of the imbalance mitigation approaches was investigated independently to evaluate its impact on the classification performance of the selected CNN architectures.

The performance of the models was evaluated through four commonly used evaluation metrics: accuracy, precision, recall, and F1-score. While the accuracy metric gives an overall classification performance estimation, the precision, recall, and F1-score metrics give a more detailed evaluation of the model’s performance, especially in case of highly imbalanced multi-class datasets, where recognition of the minority classes is crucial.

#### 4.2.2. Evaluation Metrics

Model performance was quantified using accuracy, precision, recall, and F1-score, all computed as macro-averages across the lesion classes so that minority classes contribute equally.

Accuracy = (TP + TN)/(TP + TN + FP + FN)
Precision = TP/(TP + FP)
Recall = TP/(TP + FN)
F1-score = 2 × (Precision × Recall)/(Precision + Recall)

The training setup used in all experiments is given in Table 2.

### 4.3. Implementation Details of Imbalance Mitigation Techniques

Additional implementation details are provided for the investigation of the class imbalance mitigation techniques to reproduce the results. Preprocessing and class balancing procedures were applied to the training dataset only after the 80%/20% train–test split. The independent test dataset was kept constant throughout all experiments conducted. Deep feature extraction was made using the pretrained CNN model before applying feature space oversampling procedures. Detailed information about the implementation of the key balancing methods is given below.

#### 4.3.1. SMOTE-Based Techniques

In the deep feature embedding space, SMOTE, ADASYN, and SMOTE-LOF techniques were used, but not in the raw dermoscopic images. The vectors of the features extracted by the pretrained CNN model served as inputs for oversampling algorithms, allowing us to generate synthetic minority samples in the low-dimensional semantic feature space instead of performing unrealistic pixel-level interpolation when generating synthetic samples in the raw image space [38,39].

#### 4.3.2. Balanced MixUp

The Balanced MixUp technique was implemented through a linear interpolation of pairs of training samples and their labels, with the mixing coefficient drawn from the Beta distribution. The interpolation parameter value was set to α = 1.0 to have the same probability for each sample during the feature interpolation process [40].

#### 4.3.3. GraphSMOTE

The GraphSMOTE algorithm was also implemented in the feature embedding space. The deep feature vectors extracted by the pretrained CNN model were represented as nodes in the graph, and the neighborhood relations were defined between samples lying in similar feature regions. Then, the synthetic minority embeddings were generated based on the interpolation in the graph.

Because GraphSMOTE was originally designed for node classification on explicitly graph-structured data, it was adapted to the grid-structured dermoscopic images by treating the problem as a graph-construction task in feature space rather than pixel space. Specifically, a k-nearest-neighbor graph (k = 5) was built over the deep feature embeddings extracted from the pretrained CNN, where each image was represented as a single graph node and edges connected samples with the highest cosine similarity within the same inority class. This converts the otherwise unstructured, grid-based image data into an explicit graph representation, on which the original GraphSMOTE edge-generator and node-interpolation steps could be applied unchanged; the resulting synthetic embeddings, rather than synthetic images, were then reintroduced into the classifier’s feature space during fine-tuning [27].

#### 4.3.4. GAN-Based Image Augmentation

To implement the GAN-based augmentation, a Deep Convolutional Generative Adversarial Network (DCGAN) was created, including the generator and discriminator networks. The generator was trained to synthesize realistic minority-class dermoscopic images, and the discriminator classified generated samples and real images during the adversarial training procedure. Finally, the generated images were incorporated into the training set to increase the number of minority-class samples before CNN fine-tuning.

The generator network consisted of a stack of transposed-convolution (deconvolution) layers that up-sampled a 100-dimensional latent noise vector into a 224 × 224 RGB image, with batch normalization and ReLU activations after each layer and a final Tanh output layer. The discriminator mirrored this structure with strided convolution layers, LeakyReLU activations (slope 0.2), and a sigmoid output classifying inputs as real or generated. The DCGAN was trained separately for each minority class using the Adam optimizer (learning rate 0.0002, β1 = 0.5), a batch size of 64, and 200 training epochs, following standard DCGAN training conventions. The quality of the generated images was assessed quantitatively using the Fréchet Inception Distance (FID) between real and generated minority-class images, and qualitatively through visual inspection by two of the authors to confirm the absence of major artifacts before the synthetic samples were added to the training set.

#### 4.3.5. CNN-Based Method

The CNN-Based Method refers to a data-level baseline in which the pretrained CNN backbone itself, rather than an external oversampling algorithm, is used to synthesize additional minority-class representations. A lightweight decoder head was attached to the penultimate feature layer of the pretrained CNN and trained to reconstruct minority-class feature vectors from perturbed copies of existing minority embeddings (Gaussian noise added in feature space). The decoder-generated feature vectors were then treated as additional minority-class training instances and reintroduced into the classifier during fine-tuning. Unlike SMOTE-family techniques, which interpolate between neighboring feature vectors, or GAN-based augmentation, which relies on adversarial training between a generator and discriminator, the CNN-Based Method relies solely on the discriminative CNN encoder and a simple reconstruction objective, making it computationally lighter but less able to capture the full diversity of the minority-class feature distribution.

#### 4.3.6. Near Pseudo-Labeling

Near Pseudo (short for Nearest-Neighbor Pseudo-Labeling) is a semi-supervised, hybrid imbalance mitigation technique that expands the minority classes using confidence-filtered pseudo-labels assigned to the nearest unlabeled or held-back training neighbors of each minority sample. For each minority-class instance, the k nearest neighbors (k = 5) in the pretrained CNN feature space were identified among the pool of training images that had been withheld from the labeled minority set. The base classifier assigned a predicted label and confidence score to each neighbor, and only neighbors whose confidence exceeded a fixed threshold (0.9) were relabeled and added to the minority-class training pool for that fold. This threshold-based, neighborhood-restricted Pseudo-Labeling reduces the risk of confirmation bias and label noise that is common in standard Pseudo-Labeling approaches, since only samples that are both spatially close to genuine minority examples and confidently classified are incorporated into training.

### 4.4. Pretrained Models

In this paper, six state-of-the-art pretrained models with various feature-capturing capabilities were used, and these models are discussed in the following subsections.

#### 4.4.1. EfficientNet-B0

The selection of EfficientNet-B0 as the CNN baseline network was motivated by its efficient architecture and lightweight computation. Although it is less parameterized than deep CNN architectures, EfficientNet-B0 offers satisfactory results for medical image classification and can be used as a proper benchmark for testing the effectiveness of various methods aimed at reducing class imbalance [10].

To examine the effect of imbalance reduction approaches on classification performance, sixteen methods of handling imbalance were separately tested on the training dataset while keeping all other experiment conditions the same. Accuracy, precision, recall, and F1-score were used for measuring the performance of classification. Table 3 presents the performance of EfficientNet-B0 under various approaches to class imbalance mitigation.

**Figure 3:**
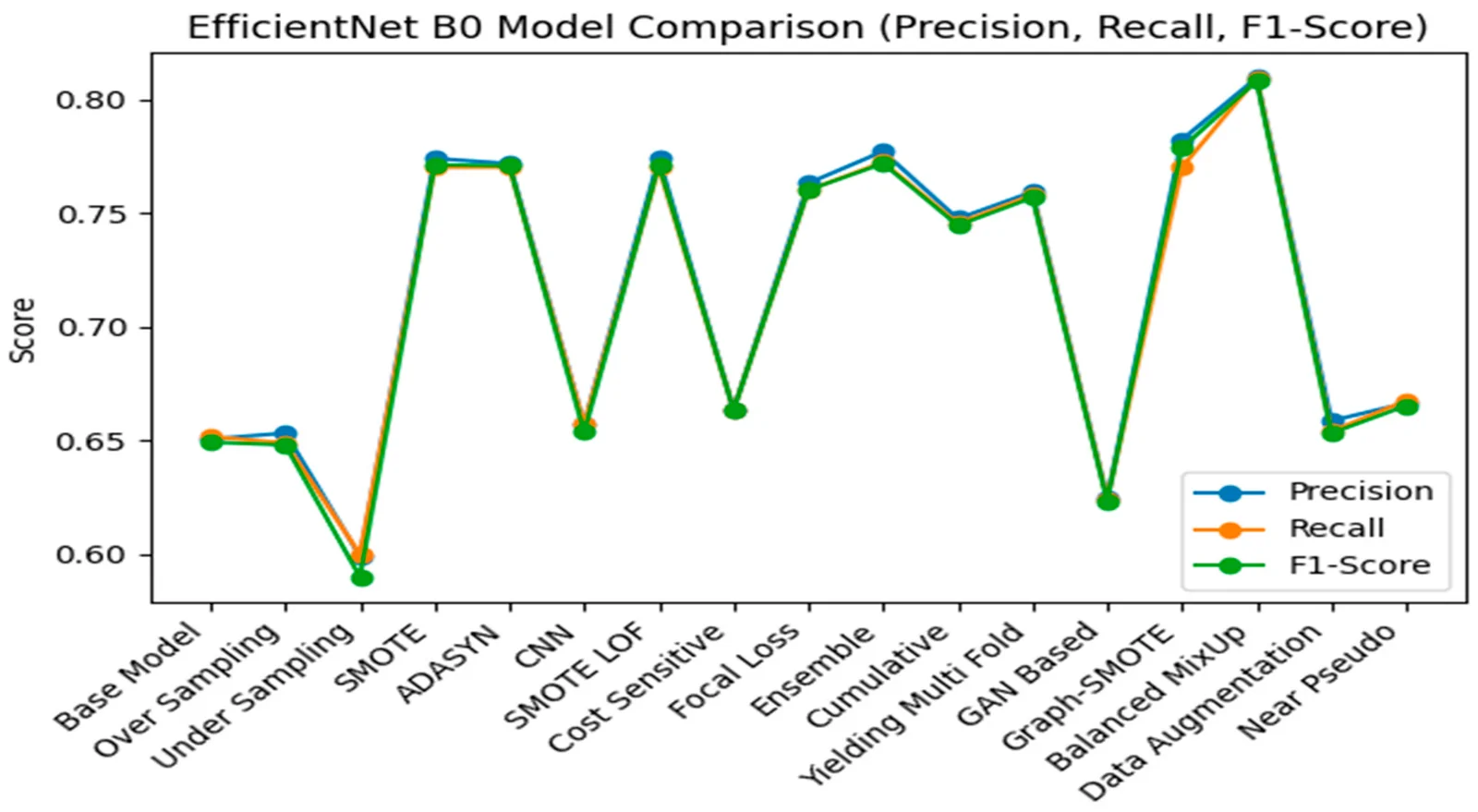
Visual representation of the accuracy, precision, recall, and F1-score of EfficientNet-B0 with different class imbalance mitigation approaches.

As shown in Table 3 and Figure 3, the performance of EfficientNet-B0 varies across the class imbalance mitigation techniques investigated. The baseline model trained on the original imbalanced dataset achieved the lowest recall and F1-score, indicating limited recognition of minority-class lesions. Traditional resampling methods, such as Random Oversampling and Random Undersampling, provided only modest improvements.

In contrast, synthetic oversampling methods, including SMOTE, ADASYN, and SMOTE-LOF, consistently improved precision, recall, and F1-score by enhancing minority-class representation during training. Among all evaluated techniques, Balanced MixUp achieved the best overall performance, producing the highest accuracy, precision, recall, and F1-score. As illustrated in Figure 3, Balanced MixUp consistently outperformed the other methods, demonstrating that advanced data-level augmentation techniques are more effective than conventional resampling methods for improving EfficientNet-B0 performance on the imbalanced ISIC 2019 dataset.

#### 4.4.2. EfficientNet-B3

EfficientNet-B3 was tested using the same experiment setting in order to study the efficacy of various methods of class imbalance reduction on a deeper neural network architecture. Since EfficientNet-B3 possesses better feature extraction abilities and a greater model capacity, the expectation is that the model will outperform lighter models in terms of classification performance. The model was tested under similar conditions to those used for training and using the sixteen class imbalance mitigation techniques. Accuracy, precision, recall, and F1-score were used as measures of performance. The classification performance of EfficientNet-B3 is shown in Table 4.

**Figure 4:**
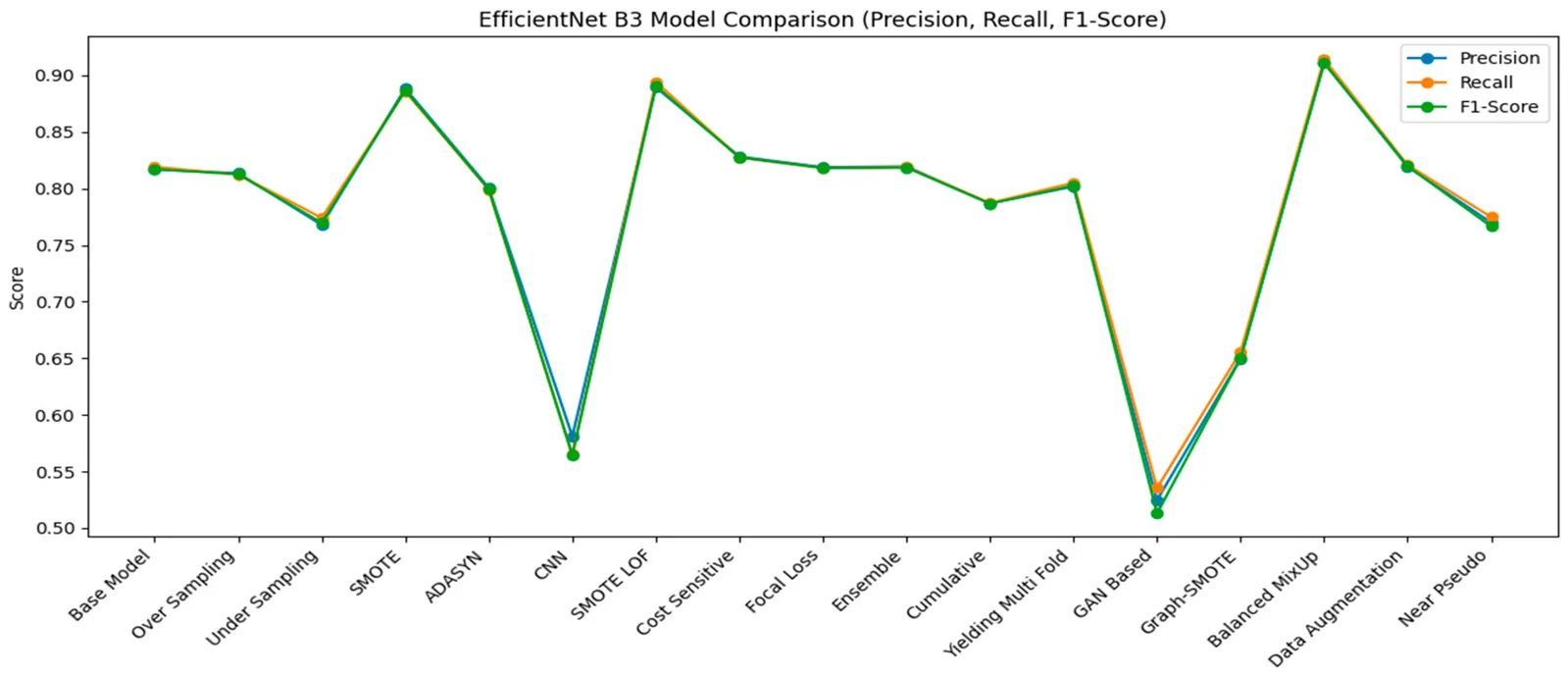
Visual representation of the precision, recall, and F1-score of EfficientNet-B3 with different class imbalance mitigation approaches.

From Table 4 and Figure 4, the performance of EfficientNet-B3 varied based on the class imbalance mitigation technique used. The baseline approach for the imbalanced dataset performed poorly compared to many of the class balancing approaches in terms of recall and F1-score values. Traditional resampling techniques like Random Oversampling and Random Undersampling did not help much in terms of performance improvement.

Synthetic oversampling techniques like SMOTE, ADASYN, and SMOTE-LOF proved beneficial for improving the classification performance. Among all tested class balancing techniques, the Balanced MixUp technique showed a superior performance in terms of achieving maximum accuracy, precision, recall, and F1-score. Figure 4 demonstrates that Balanced MixUp performed better than other class balancing techniques. Therefore, it can be inferred that Balanced MixUp is highly effective for the improvement of the classification performance of EfficientNet-B3 on the imbalanced ISIC 2019 dataset.

#### 4.4.3. ResNet50

ResNet50 was tested under the same experimental setup to examine the performance of different techniques for dealing with the problem of class imbalance on the residual learning network. Due to its unique characteristic of having residual connections, together with the capability to extract deeper features, ResNet50 is an ideal network for evaluating the influence of different balancing techniques on skin lesion detection. The performance was tested under similar experimental setup using all the sixteen imbalance mitigation techniques. The results are shown in Table 5.

**Figure 5:**
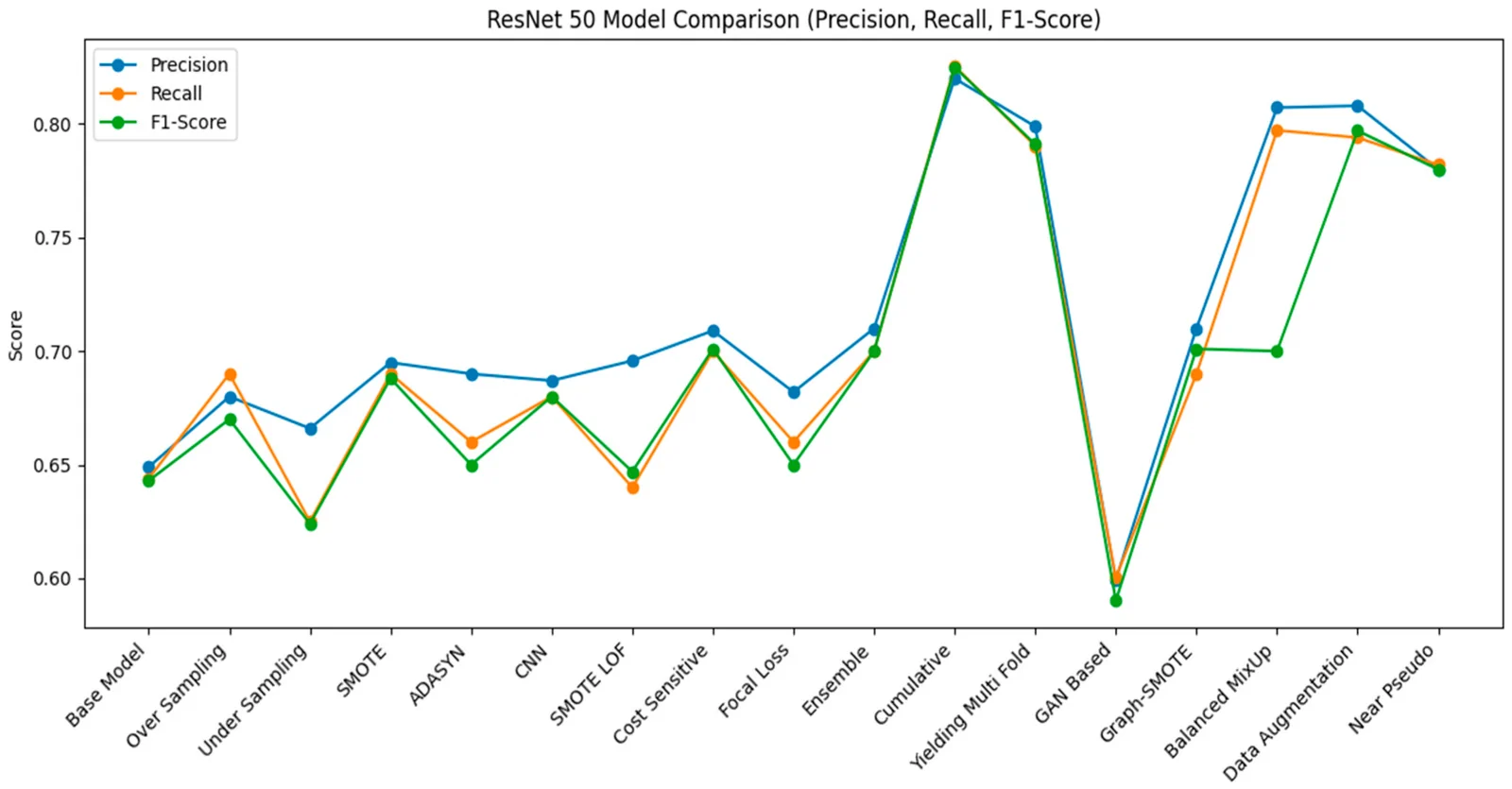
Precision, recall and F1-score comparison for ResNet50 using class imbalance mitigation techniques.

As depicted in Table 5 and Figure 5, the performance of ResNet50 depends on the class imbalance mitigation technique used. The baseline model based on the imbalanced dataset performed poorly in terms of recall and F1-score, indicating poor lesion recognition capabilities for the minority class. Traditional resampling and Cost-Sensitive Learning methods provided relatively higher performance compared to the baseline model.

Of all the tested class imbalance mitigation techniques, Cumulative Learning and Yielding Multi-Fold Training performed best in the study, achieving better precision, recall and F1-score compared to traditional resampling methods. Through their iterative process, the models helped ResNet50 learn representative features from minority-class samples, hence providing a relatively high performance. Figure 5 below compares the performance of various class imbalance techniques and demonstrates that iterative learning approaches outperform conventional resampling methods in improving the performance of ResNet50.

#### 4.4.4. InceptionV3

The InceptionV3 architecture was evaluated using the same experimental process to analyze the efficiency of various class imbalance mitigation strategies for multiscale CNN architecture. Due to the capability of the InceptionV3 architecture to derive features at several scales, the InceptionV3 is considered an ideal benchmark for skin lesion classification. InceptionV3 was tested using all sixteen class imbalance mitigation strategies in the same experimental settings. Accuracy, precision, recall, and F1-scores were used as performance measures, and their results are listed in Table 6.

**Figure 6:**
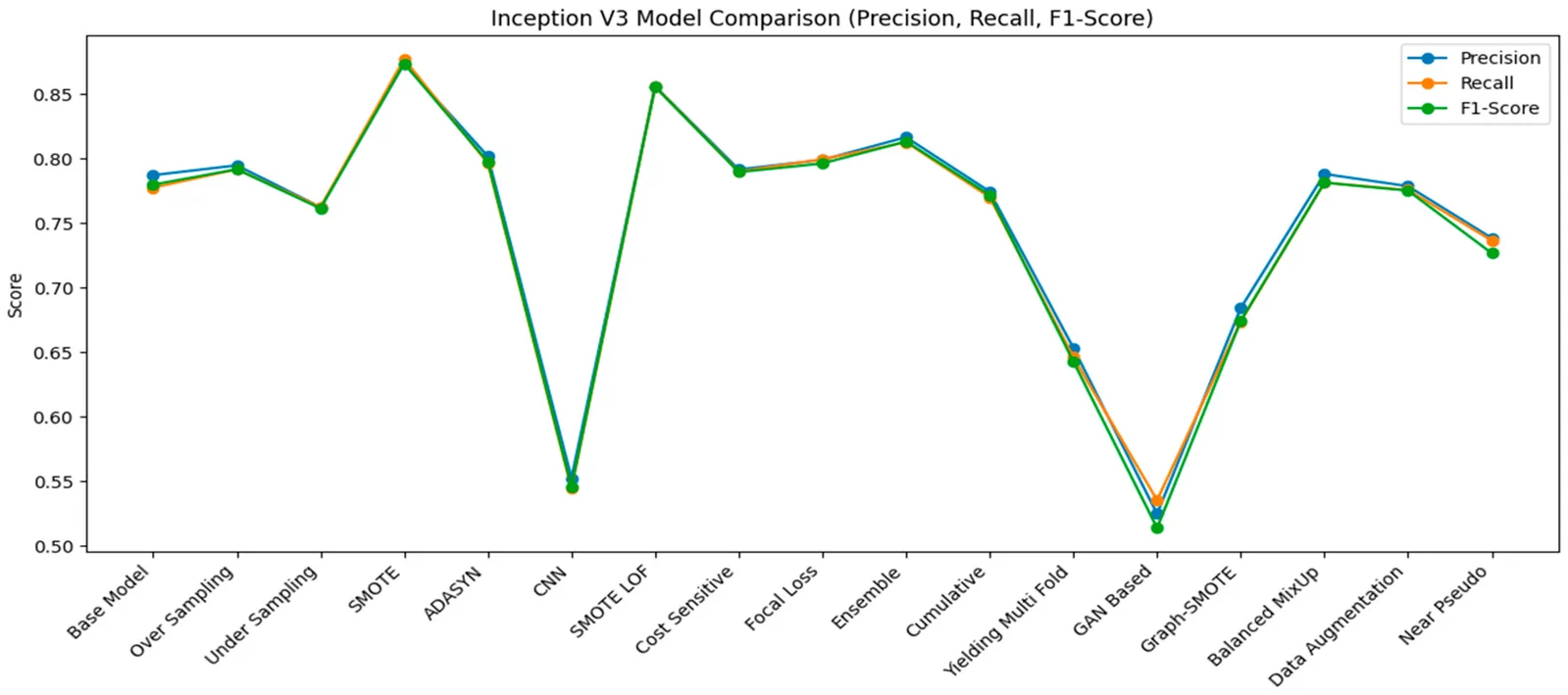
Comparing precision, recall, and F1-score of InceptionV3 with different class imbalance mitigation approaches.

As demonstrated in Table 6 and Figure 6, the InceptionV3 model performs differently depending on the applied class imbalance mitigation approach. While baseline InceptionV3 trained on the original imbalanced dataset showed a relatively low recall and F1-score, thus recognizing minority-class lesions poorly, resampling-based approaches yielded a moderate increase in comparison to the baseline model.

Among the methods considered, SMOTE, SMOTE-LOF, and ADASYN methods increased the performance of classification models through a better representation of minority-class examples during training. The SMOTE method outperformed other approaches and showed the highest accuracy, precision, recall, and F1-score. Although Balanced MixUp also outperformed the baseline model, the improvement was lower than for the EfficientNet models. Therefore, the synthetic oversampling approach is especially effective in increasing the classification performance of InceptionV3 on imbalanced ISIC 2019 dataset.

#### 4.4.5. DenseNet 121

This model was tested by conducting experiments following the same experimental methodology to test how effective the proposed methods would be for the problem of class imbalance when applied to a densely connected CNN model. The advantage of dense connection is that it allows for the reuse of features in each layer, which in turn enables efficient feature propagation. The performance of this model when tested using the sixteen different class imbalance mitigation techniques was studied under the same experimental conditions.

**Figure 7:**
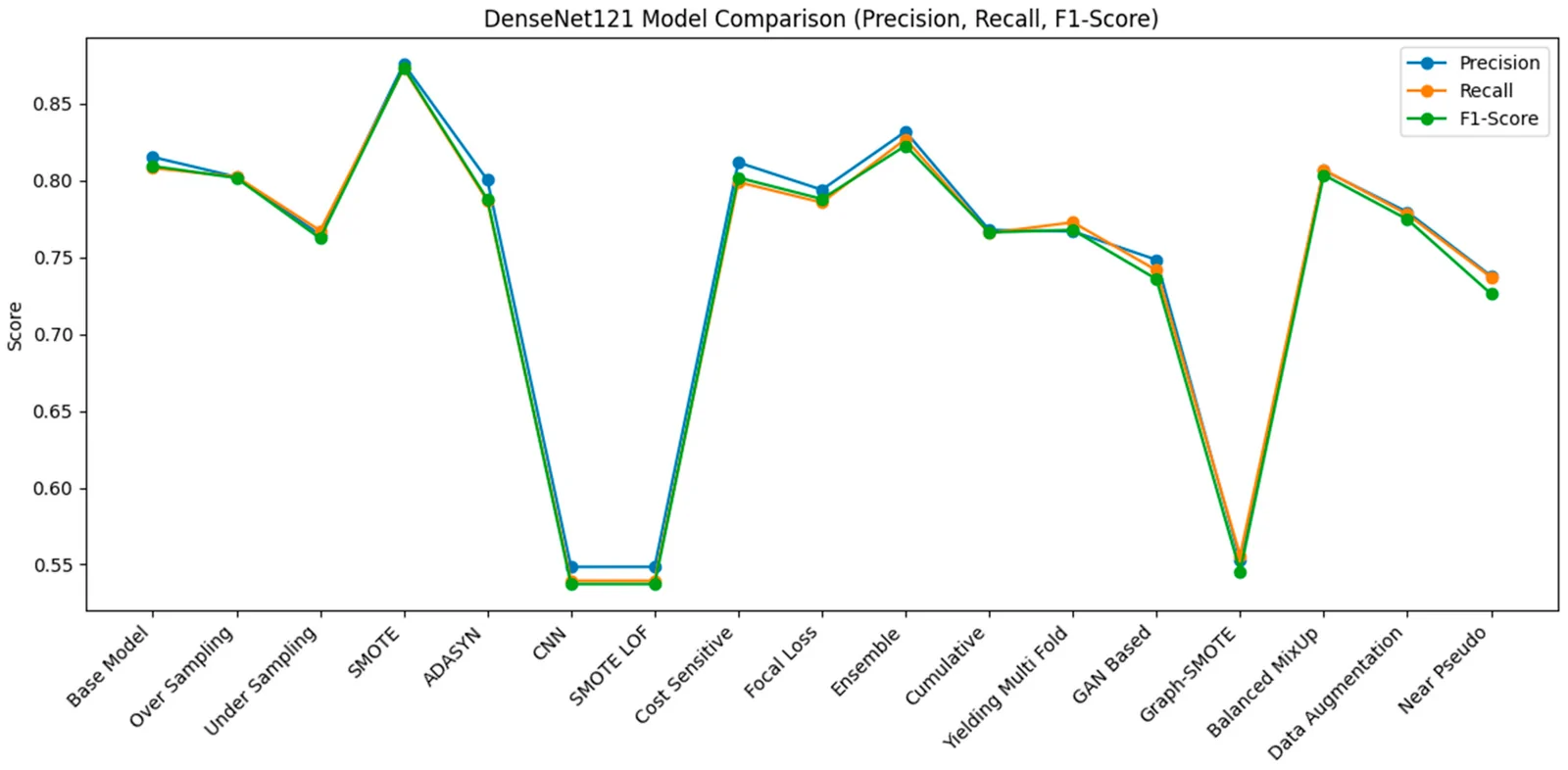
Comparison of precision, recall and F1-score of DenseNet121 for various balancing approaches.

As can be seen from the analysis provided in Table 7 and Figure 7, DenseNet121 demonstrates noticeable differences in performance between different approaches for dealing with imbalances. The network that learned from the dataset with asymmetry properties is not good at identifying the minority classes in this sample, which indicates that the network has built-in asymmetrical imbalance bias. Data generation techniques, especially SMOTE, provide the highest quality improvements, which indicates that the partial balancing of data is sufficient in the case of strong feature propagation. Ensemble and cost sensitivity methods enhance the performance by optimizing the boundaries of classification and balancing the classes. We observe that the performance of the MixUp is improved, but not as much as the approaches that use SMOTE, which indicates that DenseNet121 is able to obtain the effective representation of the minorities without aggressive interpolation of the feature space. Furthermore, with the help of visualizations like line graphs, bar charts, F1-score comparison graphs, and radar charts, one can analyze the performance and compare all the techniques explicitly, which will illustrate the difference in the performance and stability of these techniques. In general, the results of the analysis show that data balancing techniques are more effective than augmentations that are more complex in improving the performance of the DenseNet121 model.

#### 4.4.6. MobileNetV2

MobileNetV2 was evaluated using the same experimental protocol to investigate the effectiveness of different class imbalance mitigation techniques on a lightweight convolutional neural network architecture. Owing to its efficient design and reduced computational complexity, MobileNetV2 provides an appropriate benchmark for assessing the performance of imbalance mitigation strategies in resource-constrained environments. The model was trained and evaluated under identical experimental conditions using the sixteen investigated imbalance mitigation techniques. Performance was assessed using accuracy, precision, recall, and F1-score, and the corresponding results are presented in Table 8.

**Figure 8:**
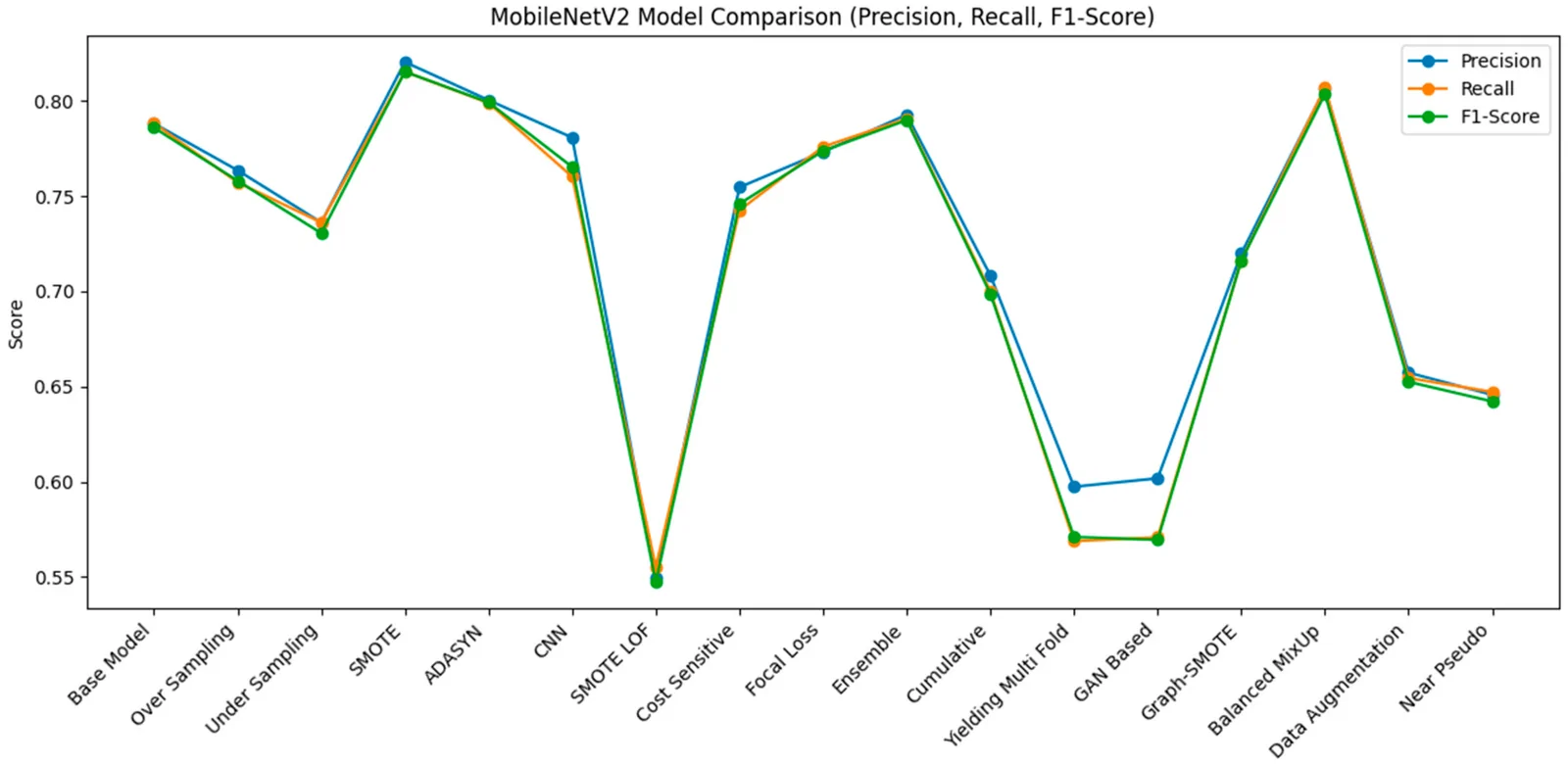
Precision, recall, and F1-score comparison between different class imbalance mitigation techniques on MobileNetV2.

From Table 8 and Figure 8, the performance of MobileNetV2 differed based on the class imbalance mitigation technique used. The baseline model built from the imbalanced dataset yielded a low recall and F1-score, which means poor detection of minority-class lesions. Resampling techniques and augmentation based on GANs showed some improvement but were not very effective compared to the baseline model.

Balanced MixUp proved to be the most effective among all tested class imbalance mitigation techniques, giving the highest accuracy, precision, recall, and F1-score. SMOTE, ADASYN, and ensemble-based techniques also showed an improvement in classification results compared with the baseline model because of the increased proportion of minority-class samples. As is shown in Figure 8, Balanced MixUp outperformed all other techniques since feature space augmentation worked especially well for lightweight architecture like MobileNetV2.

**Figure 9:**
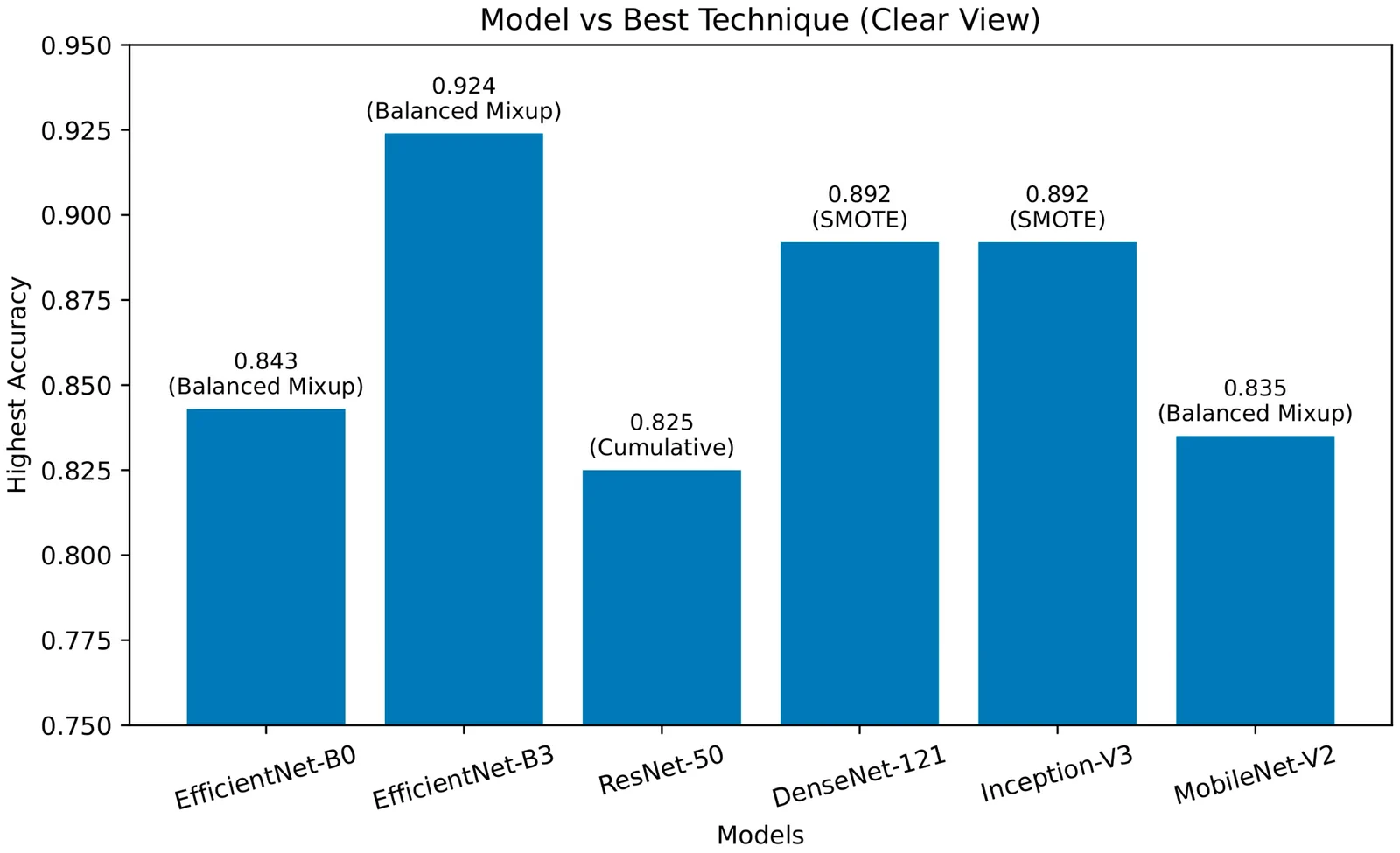
Comparison of the highest classification accuracy of the pretrained model with their best performing balancing technique.

The comparison of the highest classification accuracy achieved by different models using 17 class imbalance handling methods shown in Figure 9 indicates considerable variation in performance. EfficientNet-B3 achieved the highest accuracy of 92.4% with Balanced Mixup, making it the strongest performer among the evaluated models. DenseNet-121 and Inception-V3 both obtained an accuracy of 89.2%, with SMOTE providing their best results. EfficientNet-B0 achieved 84.3% using Balanced Mixup, while MobileNet-V2 reached 83.5%, also with Balanced Mixup. In comparison, ResNet-50 obtained the lowest accuracy of 82.5% using the Cumulative technique. Overall, the results indicate that the choice of data-balancing has a substantial effect on classification performance, with Balanced Mixup particularly effective for EfficientNet-based models, whereas SMOTE yielded the best performance for DenseNet-121 and Inception-V3 in terms of accuracy.

### 4.5. Statistical Significance Analysis

The performance of the 17 class-imbalance-handling techniques was compared across the six pretrained CNN architectures (DenseNet121, EfficientNet-B0, EfficientNet-B3, InceptionV3, MobileNetV2, and ResNet50) using the Macro F1-score reported for each technique–architecture combination in Table 3, Table 4, Table 5, Table 6, Table 7 and Table 8. Because the same 17 techniques were evaluated under the same six architectures, a non-parametric Friedman test was used to test the null hypothesis that all 17 techniques yield equivalent Macro F1 performance. Where the Friedman test indicated a significant overall difference, a Nemenyi post hoc test was applied to identify which specific pairs of techniques differed reliably.

The Friedman test statistic is computed in Equation (1) as

$$\chi_{F}^{2}=\frac{12N}{k(k+1)}\left[\sum_{j=1}^{k}R_{j}^{2}-\frac{k{\left(k+1\right)}^{2}}{4}\right]$$

where N is the number of CNN architectures (blocks), k is the number of imbalance-handling techniques (treatments), and Rj is the average rank of the j-th technique across the N architectures, with rank 1 assigned to the best-performing technique within each architecture.

The Friedman test as given in Table 9, showed a statistically significant difference among the 17 class-imbalance-handling techniques evaluated across the six pretrained CNN architectures (χ^2^ = 18.72, df = 16, *p* = 0.0022). The null hypothesis that all techniques perform equivalently is therefore rejected at the 0.05 level, indicating that the observed differences in Macro F1-score across techniques are unlikely to be due to chance alone.

As presented in Table 10, SMOTE achieved the lowest (best) average rank (3.33), followed by Balanced MixUp (3.92) and the Ensemble Method (4.42). These three techniques were consistently among the top performers across the six architectures. At the opposite end, the GAN-Based Method (16.00), Undersampling (13.33), and CNN-Based Method (13.00) obtained the highest (worst) average ranks, indicating consistently weaker Macro F1 performance relative to the other techniques.

Because the Friedman test rejected the omnibus null hypothesis, a Nemenyi post hoc test was conducted to identify which specific pairs of techniques differed significantly. The critical difference (CD) for the Nemenyi test at α = 0.05, with k = 17 techniques and N = 6 architectures, is

*CD = q_α · √(k(k +* 1*)/*6*N) =* 3.459 *· √(*17 *×* 18/36*) =* 10.08

Given the relatively small number of architectures (N = 6) relative to the number of compared techniques (k = 17), the resulting critical difference is large, so only pairwise comparisons involving techniques at the extreme ends of the rank distribution reach significance. The Nemenyi post hoc results that were statistically significant (*p* < 0.05) are reported in Table 11; all other pairwise comparisons, including SMOTE vs. Balanced MixUp and SMOTE vs. Ensemble Method, did not reach statistical significance at this sample size, despite showing consistent rank advantages in Table 10.

The only pairwise differences that survived the Nemenyi correction were those separating the best-ranked techniques (SMOTE, Balanced MixUp, and the Ensemble Method) from the worst-ranked technique, the GAN-Based Method. This indicates that while SMOTE, Balanced MixUp, and the Ensemble Method are reliable and significantly better than the GAN-Based Method, the remaining pairwise differences among the mid-ranked techniques, although suggestive of a performance ordering, cannot be distinguished with statistical confidence given the number of architectures tested.

**Figure 10:**
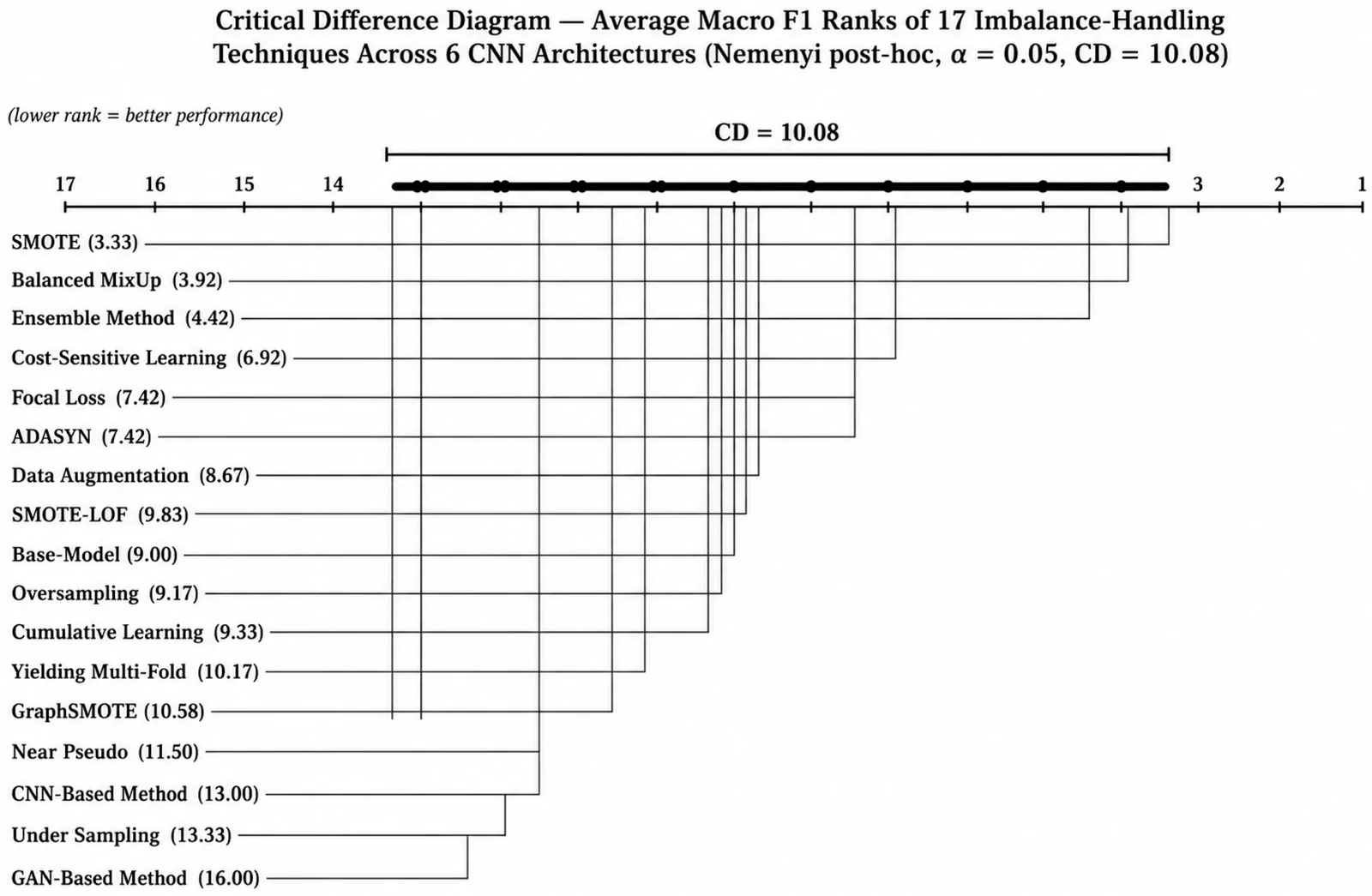
Critical difference (CD) diagram summarizing the average Friedman ranks of the 17 class-imbalance-handling techniques across six CNN architectures (Nemenyi post hoc test, α = 0.05, CD = 10.08). Techniques connected by the horizontal bar are not significantly different from the top-ranked technique.

Taken together, the Friedman and Nemenyi results confirm that the choice of class-imbalance-handling strategy has a statistically significant effect on Macro F1-score across the six pretrained CNN architectures (χ^2^ = 18.72, df = 16, *p* = 0.0022). SMOTE, Balanced MixUp, and the Ensemble Method consistently occupied the top three average ranks as shown in Figure 10 and were the only techniques whose superiority over the weakest performer, the GAN-Based Method, reached statistical significance under the Nemenyi post hoc procedure. The remaining techniques form a broad statistically indistinguishable middle band, and readers should therefore interpret rank differences among them (e.g., Cost-Sensitive Learning vs. Focal Loss vs. ADASYN) as descriptive rather than statistically confirmed, given the limited number of architectures (N = 6) available for the omnibus comparison.

The dependence of imbalance mitigation effectiveness on the underlying CNN architecture observed throughout can be attributed to differences in model capacity, receptive-field design, and feature embedding geometry across the six networks. Architectures with larger representational capacity and richer feature hierarchies, such as EfficientNet-B3, are better able to exploit the smoother, class-aware decision boundaries produced by feature space augmentation methods like Balanced MixUp and SMOTE, since these methods rely on a feature space that already separates classes reasonably well before augmentation is applied. In contrast, architectures such as ResNet50, whose residual connections favor an incremental, staged refinement of feature representations, respond more strongly to iterative and curriculum-based algorithm-level strategies such as Cumulative Learning and Yielding Multi-Fold Training, which progressively adjust the training signal rather than altering the feature distribution directly. Lightweight architectures with fewer parameters, such as MobileNetV2, have a more limited capacity to model the complex synthetic distributions introduced by GAN-based or graph-based oversampling, which explains their comparatively weaker gains from those techniques relative to simpler resampling or loss-reweighting approaches. These architectural differences in capacity and inductive bias, rather than any property of the imbalance mitigation techniques alone, explain why no single balancing strategy was optimal across all six CNN architectures.

### 4.6. Per-Class Performance of the Best Configurations

Table 12 presents a detailed analysis of the F1-scores for each class achieved by the EfficientNet-B3 model under three training configurations: The Base Model, SMOTE, and Balanced MixUp. This analysis complements the architecture-level and overall performance comparisons presented above by examining the effect of imbalance-handling strategies on individual skin-lesion categories. In particular, the table contrasts the baseline EfficientNet-B3 model with SMOTE and Balanced MixUp used with EfficientNet-B3. The per-class results highlight the differences in classification performance across both common and relatively underrepresented lesion categories. This comparison provides further insight into how the selected imbalance-handling strategies affect class-specific performance and demonstrates whether the improvements observed in the overall evaluation are consistently reflected across individual skin-lesion classes.

## 5. Conclusions

Imbalance between the classes is one of the main problems affecting the recognition efficiency of minor lesion classes during the process of medical image classification. This paper suggests a benchmarking framework which allowed us to estimate the effectiveness of sixteen techniques for class imbalance mitigation in six pretrained CNN architectures using the ISIC 2019 skin lesion dataset. All the experiments were carried out under identical conditions for the training models in order to make a fair comparison of all the discussed balancing methods.

It is evident that the effectiveness of class imbalance mitigation depends on the type of CNN architecture used. The traditional resampling methods were rather inefficient, but synthetic oversampling and hybrid learning helped achieve quite good results. From all the possible combinations of algorithms and architectures, the best results were obtained using Balanced MixUp with EfficientNet-B3—with a 92.39% accuracy, 93.30% precision, 91.36% recall and 92.33% F1-score. On the other hand, ResNet50 demonstrated good results when iterative learning strategies, such as Cumulative Learning and Yielding Multi-Fold Training, were applied. It can be concluded that different CNN architectures require different methods of class balance adjustment.

Thus, the results prove that combining an optimal CNN architecture with the proper method of class imbalance mitigation is much more efficient than the application of one balancing strategy for all types of networks. The suggested benchmarking framework serves as a practical example of how to develop reliable deep learning systems for medical image classification.

Further research should focus on investigating adaptive balance correction strategies, transformer architectures and self-supervised learning approaches.

## Figures and Tables

**Table 1:** Distribution of skin lesion classes in the ISIC 2019 dataset.

Lesion Class	Abbreviation	Number of Images	Percentage (%)
Melanocytic Nevus	NV	12,875	50.83
Melanoma	MEL	4522	17.85
Basal Cell Carcinoma	BCC	3323	13.12
Benign Keratosis	BKL	2624	10.36
Actinic Keratosis	AK	867	3.42
Squamous Cell Carcinoma	SCC	628	2.48
Vascular Lesions	VASC	253	1.00
Dermatofibroma	DF	239	0.94
Total	—	25,331	100.00

**Table 2:** Training configuration used across all experiments for all six pretrained CNN architectures and all sixteen imbalance mitigation techniques.

Parameter	Value
Optimizer	Adam
Initial Learning Rate	0.0001 (1 × 10^−4^)
Learning Rate Scheduler	None
Batch Size	32
Number of Epochs	10
Loss Function	CrossEntropyLoss
Input Image Size	224 × 224 pixels
Train/Test Split	80%/20%
Cross-Validation	3-fold stratified cross-validation
Data Augmentation	Random Horizontal Flip, random rotation (10°), color jitter
Image Normalization	ImageNet mean and standard deviation
Deep Learning Framework	PyTorch 2.10.0+cu128
Computing Device	CUDA-enabled GPU (or CPU when GPU resources were unavailable)

**Table 3:** Performance of EfficientNet-B0 using different class imbalance mitigation techniques.

Imbalance Handling Technique	Accuracy	Precision	Recall	F1-Score
Base Model	0.6516 ± 0.0041	0.6506 ± 0.0041	0.6516 ± 0.0041	0.6492 ± 0.0041
Oversampling	0.6489 ± 0.0041	0.6532 ± 0.0041	0.6489 ± 0.0041	0.6480 ± 0.0040
Undersampling	0.6006 ± 0.0038	0.5991 ± 0.0038	0.6000 ± 0.0038	0.5900 ± 0.0037
SMOTE	0.7700 ± 0.0048	0.7739 ± 0.0048	0.7700 ± 0.0048	0.7711 ± 0.0048
ADASYN	0.7705 ± 0.0048	0.7716 ± 0.0048	0.7700 ± 0.0048	0.7706 ± 0.0048
CNN-Based Method	0.6570 ± 0.0041	0.6572 ± 0.0041	0.6570 ± 0.0041	0.6537 ± 0.0041
SMOTE-LOF	0.7742 ± 0.0049	0.7758 ± 0.0049	0.7735 ± 0.0049	0.7746 ± 0.0049
Cost-Sensitive Learning	0.6636 ± 0.0042	0.6636 ± 0.0042	0.6636 ± 0.0042	0.6634 ± 0.0042
Focal Loss	0.7621 ± 0.0048	0.7630 ± 0.0048	0.7600 ± 0.0048	0.7600 ± 0.0048
Ensemble Method	0.7723 ± 0.0049	0.7770 ± 0.0049	0.7723 ± 0.0049	0.7717 ± 0.0049
Cumulative Learning	0.7454 ± 0.0047	0.7474 ± 0.0047	0.7454 ± 0.0047	0.7445 ± 0.0047
Yielding Multi-Fold	0.7578 ± 0.0048	0.7592 ± 0.0048	0.7578 ± 0.0048	0.7567 ± 0.0048
GAN-Based Method	0.6242 ± 0.0039	0.6247 ± 0.0039	0.6242 ± 0.0039	0.6234 ± 0.0039
GraphSMOTE	0.7777 ± 0.0049	0.7821 ± 0.0049	0.7700 ± 0.0048	0.7785 ± 0.0049
Balanced MixUp	0.8430 ± 0.0053	0.8093 ± 0.0051	0.8091 ± 0.0051	0.8079 ± 0.0051
Data Augmentation	0.6540 ± 0.0041	0.6586 ± 0.0041	0.6540 ± 0.0041	0.6532 ± 0.0041
Near Pseudo	0.6674 ± 0.0042	0.6664 ± 0.0042	0.6674 ± 0.0042	0.6652 ± 0.0042

**Table 4:** Performance of EfficientNet-B3 using different class imbalance mitigation techniques.

Imbalance Handling Technique	Accuracy	Precision	Recall	F1-Score
Base Model	0.8489 ± 0.0053	0.8167 ± 0.0051	0.8191 ± 0.0051	0.8171 ± 0.0051
Oversampling	0.8457 ± 0.0053	0.8135 ± 0.0051	0.8123 ± 0.0051	0.8126 ± 0.0051
Undersampling	0.8049 ± 0.0050	0.7678 ± 0.0048	0.7740 ± 0.0048	0.7696 ± 0.0048
SMOTE	0.9039 ± 0.0056	0.8883 ± 0.0055	0.8859 ± 0.0055	0.8866 ± 0.0055
ADASYN	0.8333 ± 0.0052	0.8005 ± 0.0050	0.7990 ± 0.0050	0.7993 ± 0.0050
CNN-Based Method	0.6289 ± 0.0039	0.5811 ± 0.0036	0.5654 ± 0.0035	0.5644 ± 0.0035
SMOTE-LOF	0.9069 ± 0.0057	0.8894 ± 0.0055	0.8941 ± 0.0056	0.8906 ± 0.0055
Cost-Sensitive Learning	0.8573 ± 0.0054	0.8282 ± 0.0052	0.8274 ± 0.0052	0.8274 ± 0.0052
Focal Loss	0.8498 ± 0.0053	0.8188 ± 0.0051	0.8181 ± 0.0051	0.8180 ± 0.0051
Ensemble Method	0.8509 ± 0.0053	0.8195 ± 0.0051	0.8189 ± 0.0051	0.8185 ± 0.0051
Cumulative Learning	0.8219 ± 0.0051	0.7868 ± 0.0049	0.7872 ± 0.0049	0.7866 ± 0.0049
Yielding Multi-Fold	0.8342 ± 0.0052	0.8017 ± 0.0050	0.8050 ± 0.0050	0.8026 ± 0.0050
GAN-Based Method	0.5353 ± 0.0034	0.5247 ± 0.0033	0.5353 ± 0.0034	0.5130 ± 0.0032
GraphSMOTE	0.6840 ± 0.0043	0.6496 ± 0.0041	0.6556 ± 0.0041	0.6499 ± 0.0041
Balanced MixUp	0.9239 ± 0.0058	0.9112 ± 0.0057	0.9142 ± 0.0055	0.9110 ± 0.0057
Data Augmentation	0.8498 ± 0.0053	0.8192 ± 0.0051	0.8207 ± 0.0051	0.8197 ± 0.0051
Near Pseudo	0.7963 ± 0.0050	0.7698 ± 0.0048	0.7747 ± 0.0048	0.7665 ± 0.0048

**Table 5:** Performance of ResNet50 under different class imbalance mitigation techniques.

Technique	Accuracy	Precision	Recall	F1-Score
Base Model	0.6980 ± 0.0044	0.6490 ± 0.0041	0.6440 ± 0.0040	0.6410 ± 0.0030
Oversampling	0.7000 ± 0.0044	0.6800 ± 0.0043	0.6900 ± 0.0043	0.6700 ± 0.0042
UnderSampling	0.6250 ± 0.0039	0.6660 ± 0.0042	0.6250 ± 0.0039	0.6240 ± 0.0039
SMOTE	0.6919 ± 0.0043	0.6950 ± 0.0043	0.6900 ± 0.0043	0.6880 ± 0.0043
ADASYN	0.6600 ± 0.0041	0.6900 ± 0.0043	0.6600 ± 0.0041	0.6500 ± 0.0041
CNN-Based Method	0.6820 ± 0.0043	0.6870 ± 0.0043	0.6800 ± 0.0042	0.6800 ± 0.0042
SMOTE-LOF	0.6408 ± 0.0040	0.6958 ± 0.0043	0.6400 ± 0.0040	0.6470 ± 0.0040
Cost-Sensitive Learning	0.7000 ± 0.0044	0.7090 ± 0.0044	0.7000 ± 0.0044	0.7010 ± 0.0044
Focal Loss	0.6669 ± 0.0042	0.6820 ± 0.0043	0.6600 ± 0.0041	0.6500 ± 0.0041
Ensemble Method	0.7000 ± 0.0044	0.7100 ± 0.0044	0.7000 ± 0.0044	0.7000 ± 0.0044
Cumulative Learning	0.8250 ± 0.0052	0.8200 ± 0.0051	0.8255 ± 0.0052	0.8250 ± 0.0052
Yielding Multi-Fold	0.7903 ± 0.0050	0.7989 ± 0.0050	0.7903 ± 0.0050	0.7910 ± 0.0050
GAN-Based Method	0.6003 ± 0.0038	0.5994 ± 0.0038	0.6003 ± 0.0038	0.5902 ± 0.0037
GraphSMOTE	0.6900 ± 0.0043	0.7100 ± 0.0044	0.6900 ± 0.0043	0.7010 ± 0.0044
Balanced MixUp	0.7900 ± 0.0050	0.8072 ± 0.0051	0.7972 ± 0.0050	0.7000 ± 0.0044
Data Augmentation	0.7940 ± 0.0050	0.8080 ± 0.0051	0.7940 ± 0.0058	0.7970 ± 0.0050
Near Pseudo	0.7825 ± 0.0049	0.7800 ± 0.0049	0.7820 ± 0.0049	0.7800 ± 0.0049

**Table 6:** InceptionV3 performance with different class imbalance mitigation strategies.

Technique	Accuracy	Precision	Recall	F1-Score
Base Model	0.8190 ± 0.0052	0.7873 ± 0.0050	0.7776 ± 0.0049	0.7799 ± 0.0049
Oversampling	0.8253 ± 0.0052	0.7949 ± 0.0050	0.7918 ± 0.0050	0.7918 ± 0.0050
Undersampling	0.7979 ± 0.0050	0.7625 ± 0.0048	0.7625 ± 0.0048	0.7615 ± 0.0048
SMOTE	0.8917 ± 0.0056	0.8739 ± 0.0055	0.8773 ± 0.0055	0.8740 ± 0.0055
ADASYN	0.8314 ± 0.0052	0.8019 ± 0.0050	0.7965 ± 0.0050	0.7976 ± 0.0050
CNN-Based Method	0.6101 ± 0.0038	0.5524 ± 0.0035	0.5447 ± 0.0034	0.5455 ± 0.0034
SMOTE-LOF	0.8777 ± 0.0055	0.8562 ± 0.0054	0.8560 ± 0.0054	0.8557 ± 0.0054
Cost-Sensitive Learning	0.8276 ± 0.0052	0.7917 ± 0.0050	0.7901 ± 0.0050	0.7897 ± 0.0050
Focal Loss	0.8388 ± 0.0053	0.7992 ± 0.0050	0.7996 ± 0.0050	0.7964 ± 0.0050
Ensemble Method	0.8383 ± 0.0053	0.8167 ± 0.0051	0.8128 ± 0.0051	0.8134 ± 0.0051
Cumulative Learning	0.8063 ± 0.0051	0.7745 ± 0.0048	0.7701 ± 0.0048	0.7718 ± 0.0048
Yielding Multi-Fold	0.6461 ± 0.0041	0.6532 ± 0.0041	0.6461 ± 0.0041	0.6428 ± 0.0040
GAN-Based Method	0.5353 ± 0.0034	0.5247 ± 0.0033	0.5353 ± 0.0034	0.5138 ± 0.0032
GraphSMOTE	0.6738 ± 0.0043	0.6842 ± 0.0043	0.6738 ± 0.0043	0.6743 ± 0.0043
Balanced MixUp	0.8172 ± 0.0052	0.7882 ± 0.0050	0.7815 ± 0.0049	0.7815 ± 0.0049
Data Augmentation	0.8120 ± 0.0051	0.7787 ± 0.0049	0.7758 ± 0.0049	0.7753 ± 0.0049
Near Pseudo	0.7637 ± 0.0048	0.7385 ± 0.0045	0.7366 ± 0.0046	0.7268 ± 0.0045

**Table 7:** Performance of DenseNet121 using different class imbalance mitigation techniques.

Technique	Accuracy	Precision	Recall	F1-Score
SMOTE	0.8924 ± 0.0056	0.8759 ± 0.0055	0.8730 ± 0.0055	0.8736 ± 0.0055
Ensemble Method	0.8575 ± 0.0054	0.8319 ± 0.0052	0.8270 ± 0.0052	0.8227 ± 0.0052
Base Model	0.8441 ± 0.0053	0.8153 ± 0.0051	0.8081 ± 0.0051	0.8094 ± 0.0051
Cost-Sensitive Learning	0.8369 ± 0.0053	0.8118 ± 0.0051	0.7990 ± 0.0050	0.8019 ± 0.0051
Balanced MixUp	0.8351 ± 0.0053	0.8066 ± 0.0051	0.8070 ± 0.0051	0.8037 ± 0.0051
Oversampling	0.8314 ± 0.0052	0.8024 ± 0.0051	0.8025 ± 0.0051	0.8017 ± 0.0051
ADASYN	0.8290 ± 0.0052	0.8004 ± 0.0050	0.7867 ± 0.0050	0.7878 ± 0.0050
Focal Loss	0.8219 ± 0.0052	0.7940 ± 0.0050	0.7857 ± 0.0049	0.7880 ± 0.0050
Data Augmentation	0.8108 ± 0.0051	0.7795 ± 0.0049	0.7780 ± 0.0049	0.7747 ± 0.0049
Cumulative Learning	0.8070 ± 0.0051	0.7678 ± 0.0048	0.7658 ± 0.0048	0.7663 ± 0.0048
Yielding Multi-Fold	0.8063 ± 0.0051	0.7669 ± 0.0048	0.7728 ± 0.0049	0.7678 ± 0.0048
Under Sampling	0.7923 ± 0.0050	0.7647 ± 0.0048	0.7674 ± 0.0048	0.7624 ± 0.0048
GAN-Based Method	0.7780 ± 0.0049	0.7484 ± 0.0047	0.7416 ± 0.0047	0.7358 ± 0.0046
Near Pseudo	0.7589 ± 0.0048	0.7379 ± 0.0046	0.7370 ± 0.0046	0.7262 ± 0.0046
CNN-Based Method	0.6038 ± 0.0038	0.5484 ± 0.0035	0.5394 ± 0.0034	0.5372 ± 0.0034
GraphSMOTE	0.5813 ± 0.0037	0.5530 ± 0.0035	0.5559 ± 0.0035	0.5456 ± 0.0034
SMOTE-LOF	0.7048 ± 0.0045	0.6484 ± 0.0038	0.6394 ± 0.0034	0.6272 ± 0.0033

**Table 8:** Performance of MobileNetV2 using different class imbalance mitigation techniques.

Imbalance Handling Technique	Accuracy	Precision	Recall	F1-Score
Base Model	0.8228 ± 0.0051	0.7882 ± 0.0049	0.7882 ± 0.0049	0.7860 ± 0.0049
Random Oversampling	0.7959 ± 0.0050	0.7636 ± 0.0047	0.7573 ± 0.0047	0.7581 ± 0.0047
Random Undersampling	0.7673 ± 0.0048	0.7362 ± 0.0046	0.7362 ± 0.0046	0.7306 ± 0.0045
SMOTE	0.8150 ± 0.0051	0.8205 ± 0.0051	0.8154 ± 0.0051	0.8154 ± 0.0051
ADASYN	0.8333 ± 0.0052	0.8005 ± 0.0050	0.7990 ± 0.0050	0.7993 ± 0.0050
CNN-Based Method	0.8043 ± 0.0050	0.7808 ± 0.0048	0.7603 ± 0.0047	0.7654 ± 0.0048
SMOTE-LOF	0.5827 ± 0.0036	0.5497 ± 0.0034	0.5551 ± 0.0035	0.5473 ± 0.0034
Cost-Sensitive Learning	0.7873 ± 0.0049	0.7547 ± 0.0047	0.7426 ± 0.0046	0.7460 ± 0.0046
Focal Loss	0.8097 ± 0.0051	0.7733 ± 0.0048	0.7760 ± 0.0048	0.7738 ± 0.0048
Ensemble Method	0.8260 ± 0.0051	0.7928 ± 0.0049	0.7907 ± 0.0049	0.7900 ± 0.0049
Cumulative Learning	0.7000 ± 0.0044	0.7084 ± 0.0044	0.7000 ± 0.0044	0.6988 ± 0.0044
Yielding Multi-Fold Training	0.6285 ± 0.0039	0.5974 ± 0.0037	0.5688 ± 0.0035	0.5710 ± 0.0035
GAN-Based Method	0.6118 ± 0.0038	0.6018 ± 0.0038	0.5708 ± 0.0036	0.5695 ± 0.0036
GraphSMOTE	0.7163 ± 0.0045	0.7199 ± 0.0045	0.7163 ± 0.0045	0.7162 ± 0.0045
Balanced MixUp	0.8351 ± 0.0052	0.8066 ± 0.0050	0.8070 ± 0.0050	0.8037 ± 0.0050
Data Augmentation	0.6545 ± 0.0041	0.6573 ± 0.0041	0.6545 ± 0.0041	0.6525 ± 0.0041
Near Pseudo	0.6472 ± 0.0041	0.6457 ± 0.0040	0.6472 ± 0.0041	0.6422 ± 0.0040

**Table 9:** Friedman test results for the comparison of class-imbalance-handling techniques (recomputed).

Statistic	Value
Performance Metric	Macro F1-Score
Number of CNN Architectures (N)	6
Number of Imbalance Handling Techniques (k)	17
Friedman Statistic (χ^2^F)	18.72
Degrees of Freedom	16
*p*-value	0.0022
Significance Level (α)	0.05
Decision	Reject H_0_ (*p* < 0.01)

**Table 10:** Average Friedman ranks of the 17 class-imbalance-handling techniques across the six CNN architectures (lower rank = better performance).

Rank Order	Imbalance Handling Technique	Average Rank	N (Architectures)
1	SMOTE	3.33	6
2	Balanced MixUp	3.92	6
3	Ensemble Method	4.42	6
4	Cost-Sensitive Learning	6.92	6
5	Focal Loss	7.42	6
6	ADASYN	7.42	6
7	Data Augmentation	8.67	6
8	SMOTE-LOF	8.83	6
9	Base Model	9.00	6
10	Oversampling	9.17	6
11	Cumulative Learning	9.33	6
12	Yielding Multi-Fold	10.17	6
13	GraphSMOTE	10.58	6
14	Near Pseudo	11.50	6
15	CNN-Based Method	13.00	6
16	Undersampling	13.33	6
17	GAN-Based Method	16.00	6

**Table 11:** Statistically significant Nemenyi post hoc pairwise comparisons (Macro F1 ranks, α = 0.05).

Technique A	Technique B	Rank Difference	Nemenyi *p*-Value	Sig. (α = 0.05)
Balanced MixUp	GAN-Based Method	12.08	0.0040	Yes
Ensemble Method	GAN-Based Method	11.58	0.0079	Yes
SMOTE	GAN-Based Method	12.67	0.0017	Yes
All other pairwise comparisons	—	—	*p* > 0.05	No

**Table 12:** Per-class F1-score comparison of EfficientNet-B3 under different training strategies.

Class	Abbreviation	Support	EfficientNet-B3 Base F1	EfficienNetB3 + Balanced MixUp F1	EfficientNet-B3 + SMOTE F1
Melanocytic Nevus	NV	795	0.970	0.984	0.972
Melanoma	MEL	519	0.991	0.995	0.989
Basal Cell Carcinoma	BCC	556	0.949	0.973	0.963
Benign Keratosis	BKL	416	0.907	0.950	0.926
Actinic Keratosis	AK	251	0.590	0.780	0.780
Squamous Cell Carcinoma	SCC	341	0.777	0.883	0.820
Vascular Lesions	VASC	369	0.836	0.915	0.810
Dermatofibroma	DF	335	0.718	0.845	0.832
Macro Average F1	—	—	0.842	0.916	0.849

## Data Availability

The data presented in this study are openly available at https://challenge.isic-archive.com/data/#2019 (accessed on 7 May 2026).

## Abbreviations

The following abbreviations are used in this manuscript:

CNN	Convolutional Neural Network
RF	Random Forest
DT	Decision Tree
SMOTE	Synthetic Minority Oversampling Technique
ADASYN	Adaptive Synthetic Sampling
SMOTE-LOF	SMOTE with Local Outlier Factor
GAN	Generative Adversarial Network
DCGAN	Deep Convolutional GAN
cGAN	Conditional GAN
BSS-GAN	Balanced Semi-Supervised GAN
DB-cGAN	Dual-Branch Conditional GAN
GraphSMOTE	Graph-Based SMOTE
CDSMOTE	Class Decomposition SMOTE
CLS	Cumulative Learning Strategy
IB Loss	Influence-Balanced Loss
RUS	Random Undersampling
ROS	Random Oversampling
RUSBoost	Random Undersampling Boosting
ISIC	International Skin Imaging Collaboration
NV	Melanocytic Nevi
BKL	Benign Keratosis-Like Lesions
CNN (baseline)	Standard Convolutional Neural Network
EfficientNet-B0	EfficientNet Baseline Model (Version B0)
EfficientNet-B3	EfficientNet Model (Version B3)
ResNet50	Residual Network (50 Layers)
DenseNet121	Densely Connected Network (121 Layers)
InceptionV3	Inception Version 3 Network
MobileNetV2	Mobile Network Version 2
MixUp	Data Augmentation via Linear Interpolation
Balanced MixUp	Class-Aware MixUp for Imbalanced Data
F1-score	Harmonic Mean of Precision and Recall
LOF	Local Outlier Factor
PCA	Principal Component Analysis
CV	Cross-Validation
K-Fold CV	K-Fold Cross-Validation
Y-MuFT	Yet Another Multi-Fold Training Technique
DL	Deep Learning
ML	Machine Learning
